# Embroidered textile sensors for real-time multiaxial force mapping in prosthetics

**DOI:** 10.1126/sciadv.aec9270

**Published:** 2026-08-14

**Authors:** Tianhao Yu, Axel González Cornejo, Hyeonseo Joo, Ziheng Wang, Yumin Dai, Edgar Bolívar-Nieto, Chi Hwan Lee

**Affiliations:** ^1^ School of Mechanical Engineering, Purdue University, West Lafayette, IN 47907, USA.; ^2^ Department of Aerospace and Mechanical Engineering, University of Notre Dame, Notre Dame, IN 46556, USA.; ^3^ Weldon School of Biomedical Engineering, Purdue University, West Lafayette, IN 47907, USA.; ^4^ School of Materials Engineering, Purdue University, West Lafayette, IN 47907, USA.; ^5^ Elmore Family School of Electrical and Computer Engineering, Purdue University, West Lafayette, IN 47907, USA.

## Abstract

Real-time monitoring of pressure and shear forces at the limb-socket interface is essential for optimizing prosthetic fit, comfort, and skin health. Shear forces, in particular, can aggravate irritation and cause ulceration near bony prominences, making their detection as important as pressure. However, current in-socket sensing systems—typically based on strain gages, piezoresistive films, or optical mechanisms—remain largely limited to normal pressure detection and often rely on socket modifications or rigid multilayered components, constraining seamless integration with the compliant limb-socket interface. Here, we present textile prosthetic sheaths with machine-embroidered sensor arrays that achieve simultaneous, real-time monitoring of normal and shear forces. The system wirelessly streams data for three-dimensional pressure mapping and incorporates an embroidered electroluminescent display for intuitive, on-body visual feedback. Testing with a transtibial prosthetic user demonstrated the ability to capture dynamic pressure and shear distributions during daily activities, with ground reaction and acceleration data providing complementary insights into gait symmetry and limb dynamics. These results establish embroidered textile sensors as a practical route to improving prosthetic fit, comfort, and gait stability and highlight their broader potential in rehabilitation robotics and human-machine interfaces.

## INTRODUCTION

Globally, more than 50 million people are living with limb amputation as a consequence of traumatic injuries, posing a substantial health burden (*1*). Proper fitting of limb prosthetic sockets is critical for comfort, mobility, and long-term tissue health (*2*–*8*). The interface between the residual limb and the prosthetic socket is subject to complex multidirectional forces as a result of anatomical mismatch, socket misalignment, and residual limb volume fluctuations, including normal pressures and shear stresses, which arise during daily activities such as standing, walking, and transitioning between postures (*9*, *10*). While excessive normal pressures are associated with tissue breakdown and discomfort, shear forces can exacerbate skin irritation and contribute to ulceration, especially in regions with bony prominences (*11*, *12*). Monitoring these forces in real time has thus become an area of intense interest for researchers and clinicians seeking to optimize socket fit and prevent secondary injuries.

Existing approaches to in-socket pressure (*13*, *14*) and shear sensing (*15*, *16*) have demonstrated promising results but also face certain challenges in practical implementation. Optical-based systems, such as fiber Bragg grating sensors (*17*) or emitter-photodetector pairs (*18*), enable simultaneous measurement of normal and shear forces while remaining immune to electromagnetic interference, but they typically rely on sophisticated light signal processing and integrated circuit (IC) chip components within the socket, which may present difficulties for integration into the confined geometries of prosthetic applications. As for strain gage–based transducers, they often require modification of the socket geometry to accommodate sensor mounting, sometimes involving recessing or surface reshaping, which can affect the natural pressure distribution and potentially impact clinical usability (*19*). Other wireless solutions using soft, battery-free platforms have provided multimodal monitoring capabilities but are generally constrained to normal pressure sensing alone (*20*–*22*). Consequently, translating these innovations into wearable, textile-based platforms for prosthetic users remains an ongoing challenge. The requirements of flexibility, washability, multiaxial detection, and compatibility with wireless systems call for novel materials and fabrication techniques.

In this study, we address these challenges by developing a fully embroidered, multiaxial capacitive sensing system capable of detecting both normal and shear forces at the limb-socket interface. The system satisfies key requirements for prosthetic integration, including flexibility, washability, multiaxial detection, and compatibility with wireless modules. By integrating the sensors with a Bluetooth-enabled data acquisition (DAQ) module and a textile-based electroluminescent (EL) display, we enable real-time, closed-loop feedback where pressure levels are visually communicated through illuminated embroidered pixels. The modular nature of the system also allows for easy scaling and deployment at anatomically relevant locations for targeted pressure mapping. In vivo validation in an individual with transtibial amputation demonstrates the system’s capacity to monitor real-time pressure and shear during daily functional tasks. This work represents a step forward in developing practical, wearable solutions for continuous biomechanical monitoring in lower-limb prosthetic users.

## RESULTS

### Device design and fabrication

**Fig. 1.**
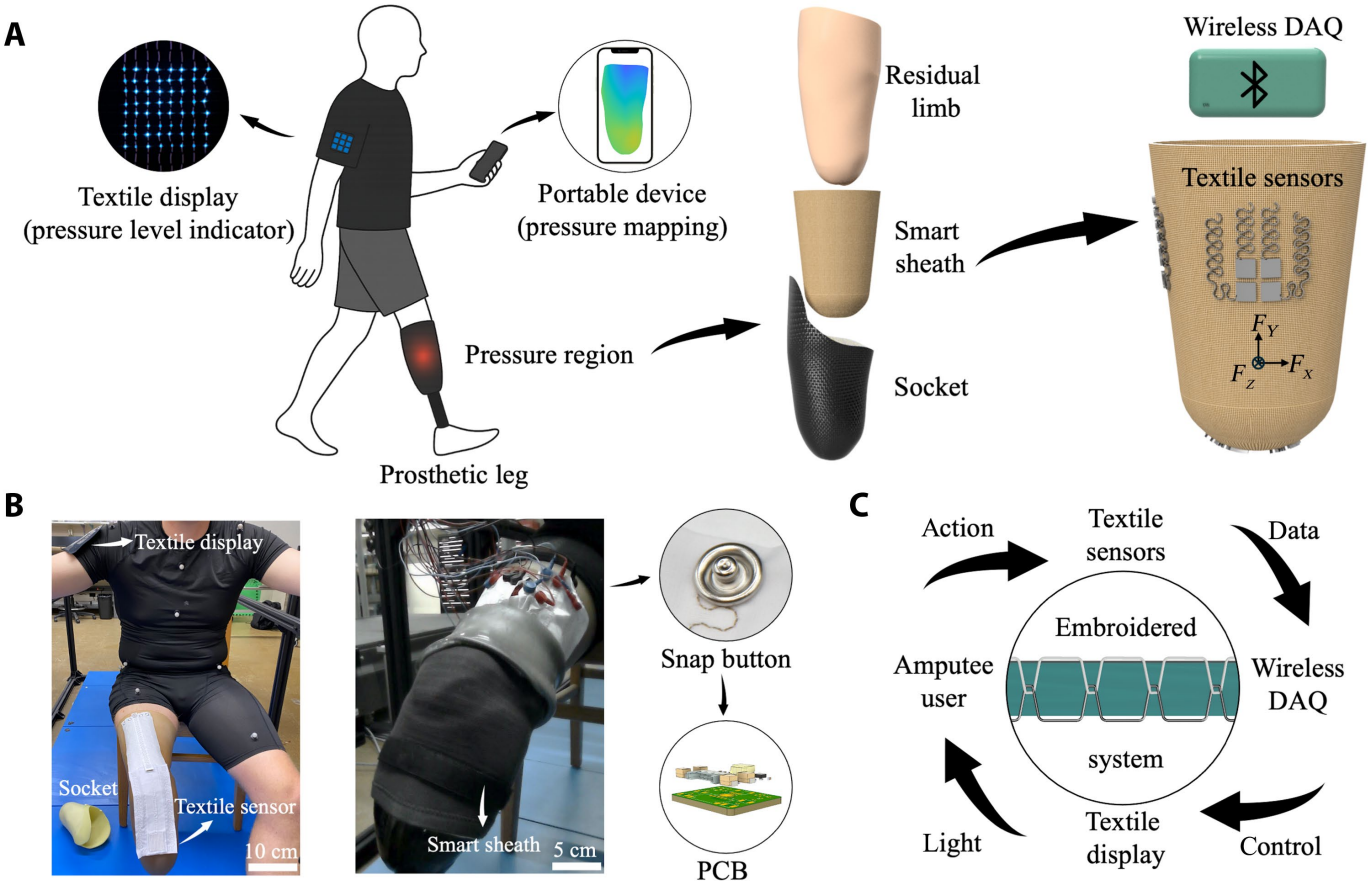
Overview of the embroidered multiaxial pressure and shear sensor for prosthetic socket interfaces. (**A**) Schematic illustration of the proposed system consisting of textile-based sensors with multiaxial sensing ability and a textile display. (**B**) Photographs of the system integrated into a wearable configuration in an individual with transtibial amputation. (**C**) The concept of an all-embroidered system.

The embroidered multiaxial pressure sensor was designed for integration into prosthetic-socket interfaces, enabling simultaneous detection of normal and shear forces (Fig. 1A). The system comprises a textile sheath embedded with the sensor array, positioned inside the prosthetic socket, and connected to a DAQ module. The modular design enables plug-and-play functionality via snap button connections to the electronics (Fig. 1B). The acquired pressure data are wirelessly transmitted to a portable device for digital mapping and simultaneously visualized in real time via an embroidered textile display located on a transtibial amputee’s sleeve. This dual-feedback approach provides both quantitative and intuitive feedback to the user (Fig. 1C).

**Fig. 2.**
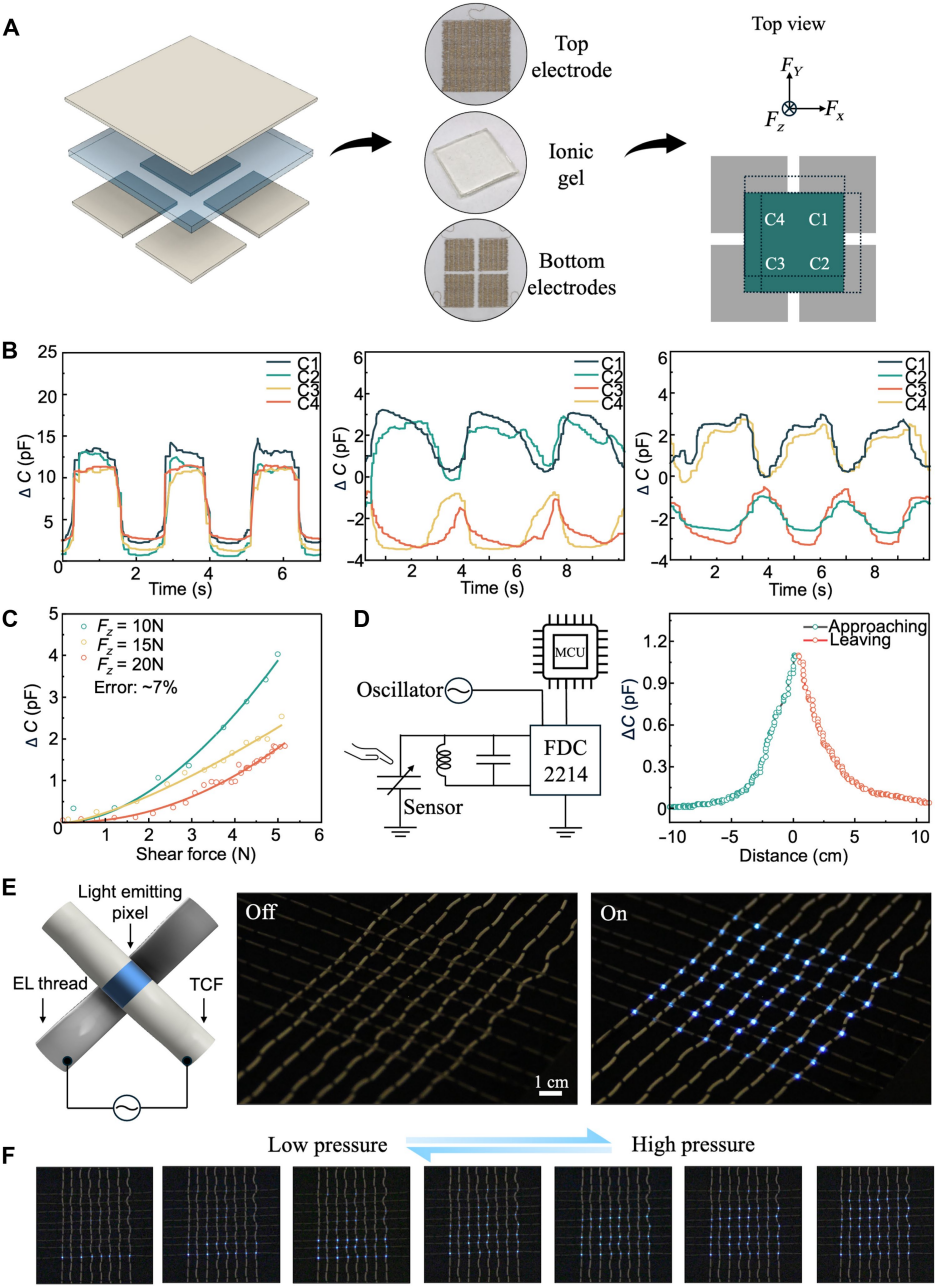
Shear pressure characterizations and an all-embroidered system. (**A**) Schematic and photographic illustration of the differential pressure sensor structure. (**B**) Relative capacitance change under external force from *Z* (*F_z_* = 10 N), *X* (*F_z_* = 10 N, *F_x_* = 5 N), *Y* (*F_z_* = 10 N, *F_Y_* = 5 N) axes, respectively. (**C**) Relative capacitance change versus the shear force *F_x_*, in the case of 10-, 15-, and 20-N applied static normal force *F_z_*. (**D**) Proximity detecting ability of the sensor. (**E**) Working mechanism of textile display and an 8 × 8 textile display under off/on state. (**F**) Textile display indicating different pressure level.

The embroidered sensor forms a multilayered capacitive structure, with a large common top electrode and four smaller bottom electrodes arranged in a quadrant configuration (Fig. 2A) (*23*–*26*). The sensor operates by detecting differential capacitance changes: Under normal compression (*F_z_*), all four quadrants exhibit similar capacitance increases, while combined normal and shear loading (*F_x_*/*F_y_* and *F_z_*) induces asymmetric changes, revealing both the magnitude and direction of shear. Circled optical images of the fabricated sensor highlight the embroidered bottom electrodes, top electrode, and an ionic gel dielectric layer. Further insights into the sensor’s material and fabrication process are provided in the Supplementary Materials (figs. S1 to S3). The sensing element is fabricated via machine embroidery (*27*–*29*) using a polyester top thread and a silver-plated conductive bobbin thread stitched into the substrate fabric (fig. S1A and movie S1). A microscopic image of the silver-plated conductive thread shows its twisted multifilament structure (fig. S1B), with tensile testing revealing a maximum load of ∼7.5 N at ∼16% strain before rupture (fig. S1C). The embroidery quality is influenced by the design parameters and machine settings. As shown in fig. S2, varying the stitch spacing from 0.2 to 0.4 mm affects the density and uniformity of the embroidered pattern, which is critical for electrical consistency. Optimal embroidery tension is also essential, as demonstrated in fig. S3: Excessive tension leads to fraying and poor coverage, whereas insufficient tension results in loose, uneven stitches. Only properly balanced tension produces uniform and well-adhered conductive patterns suitable for sensor fabrication.

### Shear pressure characterizations and an all-embroidered textile system

In Fig. 2B, the sensor can resolve relative capacitance changes (Δ*C*) under external forces applied along the *Z* (normal), *X*, and *Y* (shear) axes. In the *Z*-axis loading scenario, all four bottom electrodes (C1 to C4) show uniform Δ*C* increases due to compressive deformation of the dielectric layer. In contrast, *X*- and *Y*-axis shear loading induces differential capacitance changes among C1 to C4, reflecting relative displacement of the top electrode with respect to the quadrant bottom electrodes (movie S2).

Figure 2C further quantifies the sensor’s shear response, showing Δ*C* versus applied shear force (*F_x_*) under static normal forces (*F_z_*) of 10, 15, and 20 N, with cyclic shear test in fig. S4, showing stable mechanical integrity. Simultaneous normal and shear pressures were decoupled through a decomposition approach. The normal pressure component was calculated as the mean capacitance change across the four quadrant electrodes. The shear component was then extracted by subtracting the mean capacitance from each individual channel, thereby isolating the relative capacitance variations associated with shear deformation. The capacitance changes increase with *F_x_*. However, this effect also reflects a physical constraint common to shear-capacitive sensors: As the static normal pressure increases, the top and bottom electrodes are pressed together more tightly, which enhances frictional coupling and suppresses relative sliding motion at the interface. This phenomenon has been observed in prior works (*30*, *31*), where excessive *F_z_* caused the top electrode to lose its ability to move tangentially, effectively masking the shear response. In addition, high normal preload increases the threshold tangential force required to initiate detectable shear-induced capacitance changes. Mechanistically, preload-induced suppression can be attributed to (i) increased real contact area and interfacial frictional constraints under higher normal load, which restrict lateral deformation and reduce incremental shear-induced displacement, (ii) contact-mechanics–driven stiffening of the compressed dielectric layer, and (iii) viscoelastic effects under time-varying compressive loading (*Fz*), such as stress relaxation, which may further reduce dynamic shear contrast. Potential mitigation strategies include tuning the modulus, thickness, and microstructure of the dielectric layer to preserve lateral compliance under compression. In our sensor, this behavior suggests that while moderate normal forces facilitate better electrode contact and improve shear signal resolution, overly large *F*_*z*_ values may dominate the mechanical deformation, resulting in diminished shear sensitivity. Therefore, there exists a trade-off: Adequate *F*_*z*_ is necessary to stabilize the sensor’s baseline response, but excessive normal force constrains shear detection due to reduced electrode mobility and increased viscoelastic resistance of the dielectric layer. Reconstruction error was quantified by comparing reconstructed shear force with the applied ground-truth shear, and it remained ∼7% within the preload range corresponding to clinically relevant regions. To enable precise monitoring of small capacitance changes induced by pressure and shear at the sensor interface, we implemented a high-resolution (28-bit) four-channel capacitance-to-digital converter (FDC2214) (*32*–*34*). While this system also detects proximity-induced capacitance changes (Fig. 2D), such variations are minor compared to pressure signals and do not interfere with sensor operation (*35*, *36*). Furthermore, shielded wiring was used throughout the system to suppress environmental noise and capacitive coupling effects, ensuring reliable DAQ under in vivo conditions. We further evaluated the cross-talk and drift performance of the device. Single-channel indentation tests revealed dominant response in the actuated channel with only minor parasitic coupling among neighboring channels (fig. S5A). Under a constant 20-N load held for 60 min, the device exhibited minimal drift after an initial transient stabilization (fig. S5B).

The sensor is integrated into an all-embroidered system, where a microcontroller unit (MCU) processes capacitance signals and actuates a textile-based EL display. The working mechanism of the EL display is illustrated in Fig. 2E: Embroidered EL threads form a cross-point array with a transparent conductive fabric (TCF), generating light emission when ac voltage is applied across selected intersections (*37*, *38*).

To achieve uniform coating of EL particles and ensure consistent embroidery performance, we developed a custom automated winding and heating system adapted from our previous works (*39*, *40*). As illustrated in fig. S6A, the system comprises a stepper motor–driven winder, a direction control module, and multiple heating chambers arranged in series. Conductive thread passes sequentially through these heated zones, allowing gradual solvent evaporation during the coating process. The actual setup (fig. S6B) shows three-dimensional (3D)–printed housings. This design ensures that the solvent-based coating uniformly covers the entire thread length without aggregation or defects. The efficacy of this process is verified by thermal imaging and microscopic observation. Infrared imaging (fig. S7A) confirms the temperature distribution within the heating coil, ensuring sufficient thermal energy (∼87°C) to drive off residual solvents without damaging the conductive fibers. A close-up image (fig. S7B) demonstrates the resulting uniform coating along the thread surface after passing through the nozzle. A waterproof and transparent sealing layer (Gorilla Glue Inc.) was applied to encapsulate the TCFs, improving mechanical durability, electrical insulation, and oxidation resistance (fig. S7C). This continuous process enables high-throughput laboratory-scale fabrication (∼3 m/hour) of EL threads with excellent reproducibility, critical for reliable performance in wearable textile systems (movie S3).

This integration is demonstrated in an 8 × 8 textile display (Fig. 2E), which transitions between off and on states under MCU control. The display provides real-time feedback of pressure levels, as shown in Fig. 2F, where increasing pressure progressively illuminates more rows of the array (movie S4). Although partial ghosting artifacts were observed from the multiplexed driving architecture, the illuminated regions remained distinguishable during operation. Improved driving and addressing strategies may alleviate ghosting artifacts and yield better display contrast. This direct visual indication of interface pressure offers a highly intuitive feedback mechanism for users, supporting applications in prosthetic socket monitoring and wearable healthcare.

### Dielectric layer optimization and normal pressure characterizations

The ionic gel dielectric layer was engineered to maximize the pressure sensitivity and mechanical compliance of the embroidered pressure sensor. The precursor formulation includes benzyl acrylate (BA) as monomer, poly(ethylene glycol) diacrylate (PEGDA) as cross-linker, 2,4,6-trimethylbenzoyl-diphenylphosphine oxide (TPO) as photoinitiator, and 1-ethyl-3-methylimidazolium bis(trifluoromethylsulfonyl)imide ([EMIm][TFSI]) as ionic liquid, and poly(ethylene glycol) methyl ether methacrylate (PEGMA) to avoid the macroscopic phase separation between polymer network and ionic liquid during photopolymerization (fig. S8A). Upon ultraviolet (UV) irradiation, the photoinitiator generates free radicals that initiate polymerization, forming a cross-linked polymer network in which ionic liquid domains are uniformly distributed (fig. S8B).

The dielectric functions as an electric double layer (EDL) capacitor (fig. S8C), where mobile ions accumulate at electrode interfaces, markedly increasing the interfacial capacitance compared to conventional dielectrics (*41*–*43*). This structure allows pressure-induced deformation to alter the contact area and capacitance, enabling highly sensitive detection of normal forces.

**Fig. 3.**
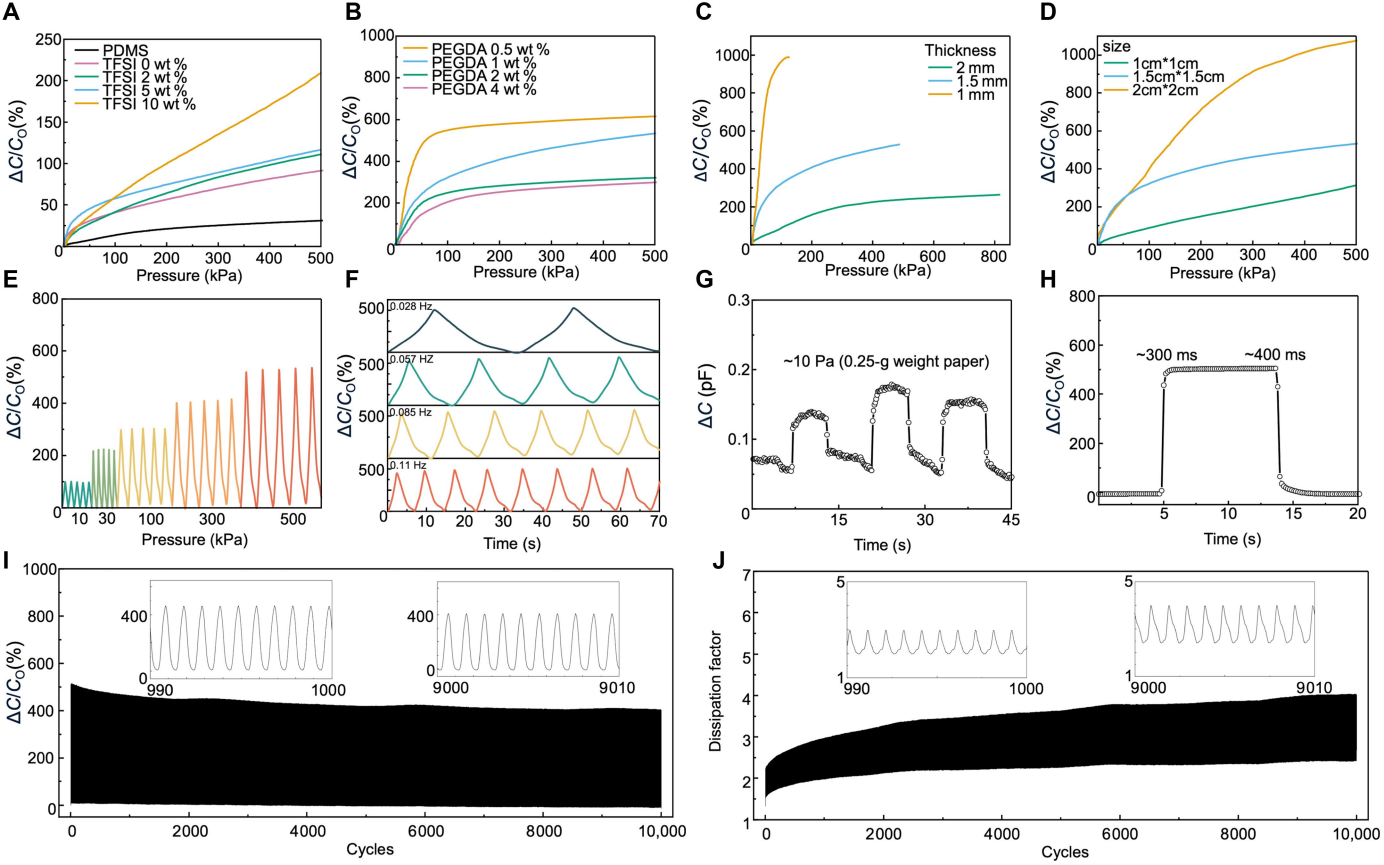
Sensing optimization and normal pressure characterizations. (**A**) Different TFSI weight percentage. PDMS, polydimethylsiloxane. (**B**) Different PEGDA weight percentage. (**C**) Different thickness. (**D**) Different size. (**E**) Cyclic relative capacitance responses of the sensor to different pressures levels. (**F**) Cyclic relative capacitance responses of the sensor to 500 kPa at different frequencies. (**G**) Lowest detection limit. (**H**) Respond and recovery time of the sensor during loading/unloading of 500 kPa. (**I**) The reliability test of the sensor through 10,000 loading/unloading cycles (0 to 500 kPa), with ∼10% peak-to-peak drift. (**J**) Dissipation factor variation during 10,000 loading/unloading cycles at a pressure of 500 kPa.

Systematic optimization of the gel composition and device geometry revealed several key trends. The experiment setup for detecting the capacitance change of the sensor is schematically shown in fig. S9. Increasing the TFSI concentration from 0 to 10 wt % (Fig. 3A) enhanced sensitivity due to greater ionic mobility and EDL formation. Notably, sensors with 10 wt % TFSI achieved relative capacitance changes (Δ*C*/*C*_0_) exceeding 200% under 500 kPa. We only tested up to 10 wt % TFSI because further increasing the ionic liquid concentration resulted in excessive ionic conductivity, effectively transforming the dielectric layer into a conductive medium. This compromised the electric double-layer formation and caused the sensor to lose its capacitive response. Varying PEGDA cross-linker content from 0.5 to 4 wt % (Fig. 3B) modulated the gel stiffness: Higher PEGDA slightly lowered sensitivity. Devices fabricated with a 1-mm-thick dielectric layer (Fig. 3C) displayed the highest Δ*C*/*C*_0_ (>800%) within 100 kPa, while thinner layers showed reduced dynamic range. Similarly, larger electrode areas (up to 2 cm by 2 cm; Fig. 3D) produced greater capacitance changes due to increased charge accumulation at the interfaces.

Cyclic loading tests reveal a broad detection range up to 500 kPa, with relative capacitance changes exceeding 500% under repeated pressure application (Fig. 3E). The sensor maintains stable response characteristics across multiple loading cycles at varying pressures, indicating minimal drift and high repeatability. Dynamic characterization under cyclic pressures at 500 kPa and different frequencies (0.028 to 0.11 Hz) confirms the sensor’s ability to follow periodic mechanical stimuli without phase lag (Fig. 3F). The distinct response profiles at different frequencies suggest reliable operation across a physiologically relevant range of actuation rates. The sensor exhibits a marked detection limit of ∼10 Pa, demonstrated by its ability to resolve the application of a 0.25-g paper (∼10 Pa) with high signal fidelity (Fig. 3G). Fast response and recovery times of ∼300 and 400 ms, respectively, are observed during loading and unloading cycles of 500 kPa (Fig. 3H), enabling real-time monitoring of transient pressure events.

Durability tests under repeated cyclic loading at 500 kPa over 10,000 cycles confirm the sensor’s mechanical robustness and electrical stability (Fig. 3I). The relative capacitance change remains consistent throughout the cycles; insets in Fig. 3I illustrate stable signal waveforms at the 1000th and 9000th cycles, underscoring excellent fatigue resistance and minimal signal degradation. Although the ionic gel dielectric layer itself exhibits some intrinsic tackiness due to the presence of chemical functional groups, a thin layer of superglue was applied between the textile electrode and the dielectric layer to ensure robust mechanical adhesion. This additional bonding step prevents potential delamination or slippage at the electrode-dielectric interface during repeated mechanical loading. Direct quantitative measurements of interfacial adhesion were not performed in this study. Potential failure modes may include delamination caused by viscoelastic mismatch between the ionic gel and textile substrate. The incorporation of encapsulation layers could help improve long-term interfacial reliability. As shown in fig. S10A, the addition of glue had negligible effect on the initial capacitance (∼85 pF), indicating that the glue layer does not interfere with the EDL formation or electrode contact. However, long-term cyclic tests revealed that samples without glue exhibited greater signal drift and increased noise under repeated pressure loading (fig. S10B). This suggests that the glue layer prevents interfacial slippage or delamination, thereby improving the mechanical reliability and signal stability of the sensor without compromising its electrical performance. We further examined the washability of the sensor, which was placed in a mesh laundry bag and underwent 30 machine washing cycles (fig. S10C). Capacitance measured after washing showed only a slight decrease (∼7%), indicating good mechanical resilience and strong adhesion.

As for the dissipation factor (Fig. 3J), it increases only modestly (from ∼2 to ∼4), which indicates a progressive increase in internal energy loss within the dielectric layer. The gradual increase could be attributed to several factors: (i) mechanical fatigue of the gel matrix, potentially causing microstructural rearrangements and reduced efficiency of ion transport pathways; (ii) viscoelastic relaxation effects of the polymeric network, which become more pronounced under repeated mechanical stress. This indicates that the sensor retains excellent electrical stability and minimal dielectric breakdown despite long-term cyclic loading. These results highlight the embroidered sensor’s potential for applications requiring high sensitivity, fast response, and long-term reliability, such as prosthetic-socket interface monitoring and wearable healthcare systems. A comparison with representative textile and flexible multiaxial sensors is provided in table S1 to contextualize the advancement of the proposed system.

### Dynamic pressure analysis in an individual with transtibial amputation

**Fig. 4.**
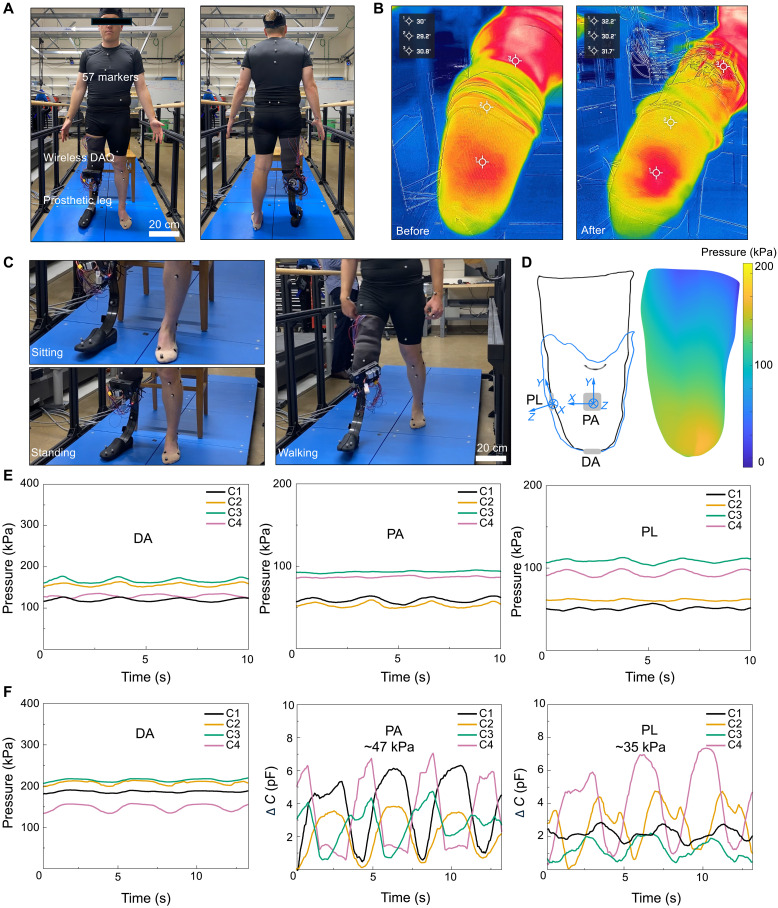
In vivo evaluation of the system in an individual with transtibial amputation. (**A**) Photographs of the participant with the embroidered sensor, textile display, and wireless DAQ system. (**B**) Thermal images of the residual limb before and after test, showing temperature changes. (**C**) Test scenarios including sitting, standing, and walking. (**D**) Schematic of sensor locations on the residual limb: distal anterior (DA), proximal anterior (PA), and proximal lateral (PL), and spatial pressure distribution at the limb-socket interface reconstructed using MATLAB interpolation based on sensor data. (**E**) Recorded normal pressures from four quadrants (C1 to C4) at DA, PA, and PL locations during sit and stand. (**F**) Simultaneous measurements of normal pressure and differential capacitance at three locations during walking, showing local shear pressure (∼47 kPa at PA and ∼35 kPa at PL).

To validate the embroidered pressure sensing system in a real-world setting, we conducted in vivo testing with a participant with transtibial amputation (Fig. 4A and movie S5). The embroidered sensor array was worn inside the prosthetic socket, with a wireless DAQ module and textile display. The sensors were calibrated before test (fig. S11). The length of interconnections was limited by the frame size of the embroidery machine, but it can be extended by using conductive Velcro (fig. S12). Real-time data can be retrieved by a customized iOS application and simultaneously stored on an SD card (fig. S13). Infrared thermal imaging showed a mild regional temperature rise after the test, which remained within a range that is unlikely to compromise residual limb comfort or induce thermal discomfort during prolonged use (Fig. 4B). The normalized capacitance remains essentially constant across the tested temperature (from 20°C up to 40°C) and relative humidity (up to ∼90%) ranges, indicating negligible environmental drift (fig. S14).

Data were collected under repeated sit-to-stand cycles and walking conditions (Fig. 4C and fig. S15). Three key sensor locations—distal anterior (DA), proximal anterior (PA), and proximal lateral (PL)—were monitored for pressure signals, DA refers to the distal end of the limb, PA refers to the front side of the residual limb near the knee, and PL refers to the outer side of the residual limb near the knee. Variations in sensor placement can alter the local preload condition and limb/sensor interface geometry, so the sensors were fixed to the inner socket wall; therefore, shear directions are defined relative to the local surface orientation. At the vertical regions (PA and PL), shear primarily corresponds to motion along the socket wall. In addition, the spatial pressure distribution at the limb-socket interface can be reconstructed using MATLAB interpolation based on the obtained sensor data (Fig. 4D). During sit-to-stand cycles, the normal pressure distributions at the three sensor locations exhibited distinct patterns (Fig. 4E). At the DA site, the pressures were relatively high (>100 kPa) across all four quadrants. This pattern likely reflects the distal end bearing of the residual limb, where the soft tissues compress against the socket’s distal cap. At the PA site and PL site, higher pressures were observed in C3 and C4, and this asymmetry may be attributed to lateral residual limb movement within the socket and uneven contact caused by the anatomical shape of the lateral wall, which can create localized stress concentrations.

**Fig. 5.**
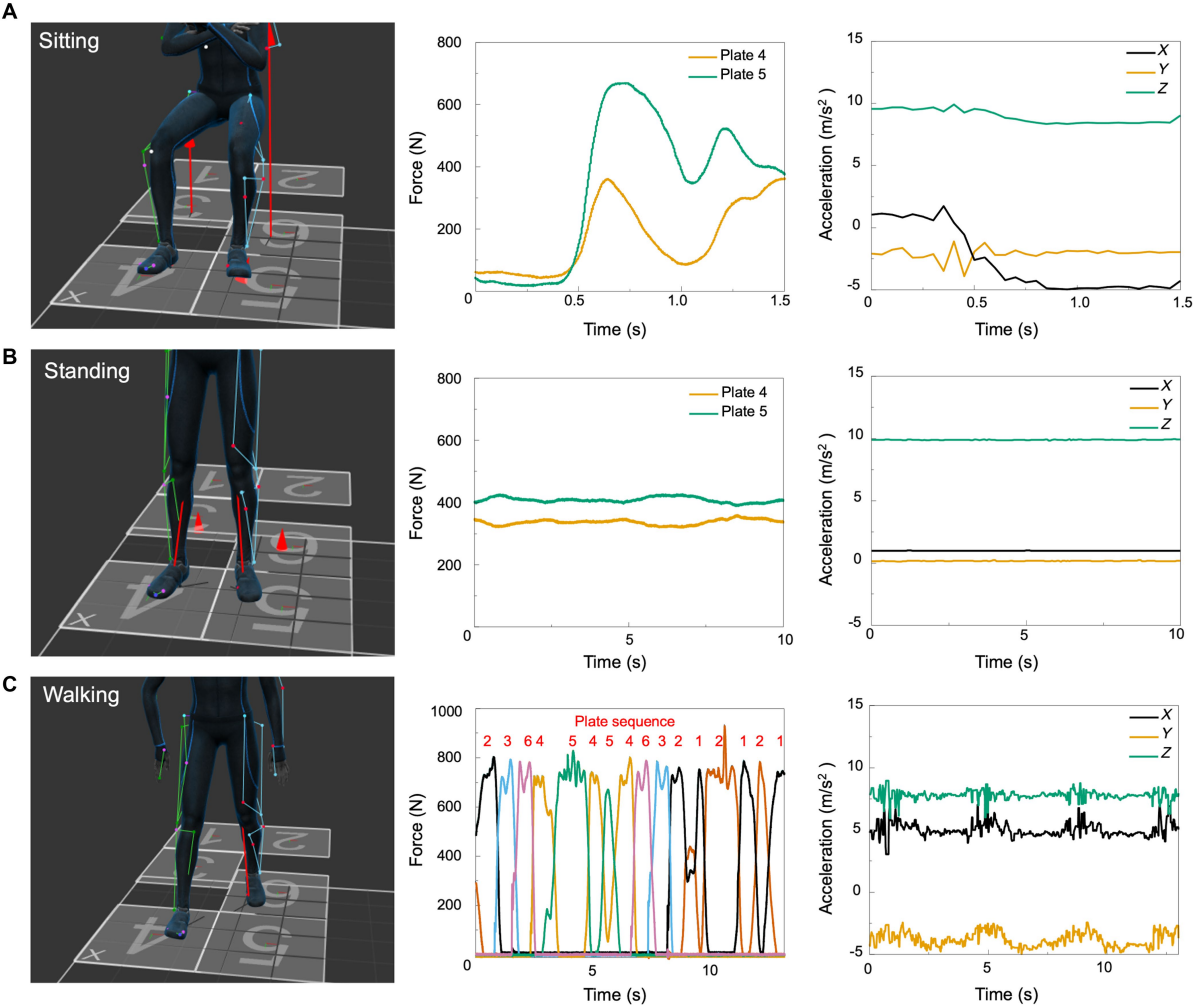
Postural analysis using force plates and motion capture. (**A**) Sit-to-stand transition: Left: Avatar showing joint trajectories and ground reaction forces. Middle: Force profiles from plate 4 (prosthetic side) and plate 5 (intact side). Right: Residual limb acceleration in three axes (*X*, *Y*, and *Z*). (**B**) Static standing. Left: Avatar during quiet standing. Middle: Force distribution showing higher load on the intact side. Right: Residual limb acceleration across all axes. (**C**) Walking. Left: Avatar during walking. Middle: Dynamic force profiles across the gait cycle, highlighting sequential plate contact. Right: Residual limb acceleration indicating periodic gait-induced fluctuations.

During walking, dynamic fluctuations in normal pressure and differential capacitance were recorded at the three sensor sites, revealing distinct loading behaviors (Fig. 4F). At the DA site, the pressure traces exhibited relatively high baseline values with oscillations across the gait cycle, and this pattern indicates that the distal limb repeatedly compresses against the socket bottom. The differential capacitance changes were minimal at this site, suggesting normal pressure domination and limited shear displacement due to strong distal anchoring of the residual limb within the socket. The PA site was accompanied by large differential capacitance changes (∼ 5 pF), which showed pronounced cyclic variations synchronized with the gait phases. This behavior reflects periodic shear pressure (∼ 47 kPa) vertically as the limb moves within the socket during swing and stance transitions. The magnitude of Δ*C* indicates significant top electrode displacement relative to the quadrant electrodes, consistent with PA sliding at the anterior socket wall. Similar to the PA site, the PL site also showed a clear gait-synchronized pattern. This likely reflects mediolateral shear pressure (∼35 kPa) horizontally due to lateral residual limb movement and minor socket gapping during dynamic loading. The signal aligns with the gait cycle timing, which is independently shown in Fig. 5 and movie S5. These findings are consistent with previously reported studies on residual limb-socket interface mechanics (*44*).

Together, these data highlight the sensor’s capability to resolve complex pressure and shear interactions at critical limb-socket interface regions during locomotion, with quadrant-level resolution capturing asymmetries in residual limb motion. It shows the influence of residual limb anatomy and socket fit on interface loading patterns, supporting its potential for monitoring residual limb-socket interface mechanics during daily activities. During testing, the volunteer wore a conventional gel sheath between the residual limb and the sensor layer, preventing direct skin contact. No noticeable discomfort or changes in wearability were reported, while clear and repeatable pressure/shear-related signals were maintained throughout gait cycles. Long-term stability of the ionic gel under dynamic microclimate conditions remains an important consideration, as solvent retention and ion migration may induce signal drift during prolonged operation. The use of more highly cross-linked polymer networks could help suppress ion migration and improve stability.

### Dynamic gait analysis in an individual with transtibial amputation

Figure 5 illustrates postural and dynamic gait analysis of the participant using synchronized force plate and motion capture data. During the sit-to-stand transition (Fig. 5A), the prosthetic side (plate 4) and intact side (plate 5) exhibited distinct ground reaction force profiles. Peak forces on the intact side reached ∼700 N, while the prosthetic side peaked at ∼400 N, reflecting a reliance on the healthy limb during this demanding transitional movement. The prominent *x*-axis acceleration observed during the sit-to-stand transition likely corresponds to a tilting motion of the prosthetic limb as the participant shifts their center of mass forward to initiate standing. Minimal changes in the *Y* and *Z* axes further suggest that the movement was predominantly sagittal without significant mediolateral or vertical perturbations.

In static standing (Fig. 5B), force measurements revealed a clear asymmetry, with the intact side supporting a consistently higher load (∼400 N) compared to the prosthetic side (∼300 N). Although healthy participants sometimes present this asymmetry, this unequal distribution implies the participant’s natural tendency to offload the prosthetic side, a common compensatory strategy in transtibial amputees. Residual limb accelerations remained low (<1 m/s^2^), indicative of stable posture. During walking (Fig. 5C), dynamic force profiles showed periodic peaks corresponding to gait phases, with sequential plate contacts identified (red annotations). Residual limb accelerations exhibited rhythmic oscillations, aligned with gait cycles. These findings collectively highlight asymmetric load sharing and movement patterns, emphasizing the need for balanced gait mechanics and enhanced comfort during prosthetic use. The gait data presented here were obtained from a single participant and are intended to demonstrate the feasibility of real-time multiaxial sensing during dynamic locomotion rather than to establish clinically generalizable asymmetry metrics. Limb loading variability and gait asymmetry are commonly observed even in healthy individuals due to limb dominance and natural biomechanical variability. Future studies involving larger cohorts will be necessary to systematically assess subject variability and clinical relevance.

## DISCUSSION

The embroidered multiaxial pressure sensing system developed in this study addresses key challenges in monitoring residual limb-socket interactions. By integrating ionic gel–based EDL capacitors with machine-embroidered electrodes, the quadrant system achieves the ability the measure both normal and shear pressure, which is critical for practical wearable applications, particularly in prosthetic users where interface forces fluctuate dynamically during daily activities. The fabric architecture can be readily tailored in size, layout, and electrode configuration to accommodate different socket shapes and sheath design. The quadrant architecture was deliberately chosen as an engineering trade-off to balance directional shear discrimination and deployment feasibility within the confined socket environment. Furthermore, the sensitivity and working range can be tuned by adjusting the dielectric thickness, modulus of the ionic gel. This tunability allows customization across different users and loading conditions, where interface pressures are generally reported to be below 350 kPa (*14*, *20*).

In vivo tests with a transtibial amputee demonstrated the sensor’s ability to capture site-specific normal and shear force distributions, revealing patterns consistent with previously reported gait asymmetries. Notably, the prosthetic side exhibited lower ground reaction forces during static standing and gait, reflecting the user’s natural compensatory strategies to offload the prosthetic limb. These findings emphasize the importance of real-time monitoring systems for assessing socket fit and informing interventions to promote balanced gait mechanics and improve wearing comfort. External biomechanical factors such as residual-limb volume fluctuations (*9*) and suspension tension variability may alter normal preload distribution within the socket and thereby influence shear sensitivity under extended wear conditions; systematic evaluation of these effects will be an important focus of future studies.

Compared with previously reported pressure sensors, our fully embroidered system combines soft, customized design with wireless DAQ and an intuitive textile display. It offers a rapid qualitative visualization tool during socket fitting and alignment, complementing the mobile application for quantitative monitoring and serving as a feedback interface with potential for richer functionality through increased pixel density. While the current method provides a simple and effective approach for decoupling normal and shear forces, limitations may arise in higher-density arrays or complex loading conditions involving nonlinear interactions and cross-talk. Advanced signal processing strategies, such as nonlinear compensation or machine learning–based approaches, could further improve multidimensional force reconstruction and enable more robust decoupling under complex conditions. In addition, integration with biomechanical models may allow estimation of localized tissue stresses, further advancing personalized prosthetic care.

## MATERIALS AND METHODS

### Materials

Benzyl acrylate [BA; stabilized with Monomethyl Ether of Hydroquinone (MEHQ); ≥97%] was purchased from TCI Chemicals (USA). PEGMA [number-average molecular weight (*M*_n_) ≈ 950], PEGDA (*M*_n_ ≈ 700), TPO, and [EMIM][TFSI] were purchased from Sigma-Aldrich (USA). All reagents were used as received without further purification.

### Machine embroidery of electrodes and textile display

Electrode patterns were designed in Autodesk AutoCAD (2019), exported as dxf format files, and then imported into Inkscape with Ink/Stitch extension for pes format file output. Last, the pes files were put into embroidery machine Brother SE1900 loaded with conductive thread (117/17 dtex 2 ply, ShopVTT) for manufacturing.

### Fabrication of ionic gel and sensor

Benzyl acrylate (BA) and PEGMA were mixed at a weight ratio of 7:3 (BA:PEGMA), calculated on the basis of the total weight of monomers excluding the cross-linker, photoinitiator, and ionic liquid components. PEGDA (1 wt %) and TPO (1 wt %) were added as the chemical cross-linker and photoinitiator, respectively. The ionic liquid [EMIM][TFSI] was incorporated at varying concentrations (0, 2, 5, and 10 wt %) relative to the total monomer content. The mixture was stirred until a clear and homogeneous precursor solution was obtained. The precursor solution was poured into a custom-designed mold and cured under UV light (365 nm) for 5 min. After polymerization, the ionic gel film was carefully demolded and laminated onto a textile-based electrode to fabricate a soft capacitive pressure sensor. This fabrication strategy was adapted with modifications from a previously reported UV-curable ionogel system.

### Fabrication of EL threads

A mixture of tetrahydrofuran (Thermo Fisher Scientific) and *N*,*N*′-dimethylformamide (Thermo Fisher Scientific) was used to dissolve thermoplastic polyurethane (TPU) pellets (Elastollan 60A, BASF). The TPU solution was then mixed with commercially available zinc sulfide (ZnS) phosphors with different dopants (Shanghai Keyan Phosphor Technology Co.) using a planetary centrifugal mixer (ARE-310, Thinky) at 2000 rpm for 5 min. The resulting TPU/ZnS phosphor mixtures were applied onto commercially available Ag-plated conductive threads by passing them through customized coating system with tip diameters of 250 μm.

### Fabrication of TCF

The commercially available transparent nylon fibers were subjected to a cleaning process involving sonication in deionized water, acetone, and isopropyl alcohol. To produce hydrophilic surfaces, the cleaned fibers were immersed in a 10 wt % resorcinol solution dispersed in ethyl acetate (Fisher Chemical) and left to dry in a hood for 10 min. The conducting layer was applied by dipping the resorcinol-coated fibers into a 0.25 wt % dispersion of silver nanowires (AgNWs) (Flexiowire 2020, SG Flexio Co.). To ensure a reliable and uniform electrical conductivity, the dip-coating process was repeated three times. The resulting TCF was air dried for 30 min after the dip-coating cycle.

### Characterizations of pressure sensor

To measure the electromechanical properties of the pressure sensor, a LCR meter (E4980AL, Keysight) and a custom-built LabView code (National Instruments) were used in a two-wire configuration, and testing was conducted using a mechanical testing machine (Mark-10, Willrich Precision Instruments).

### Fabrication of wireless DAQ device

Commercial software (Autodesk EAGLE Version 9.6.2) was used to generate schematic diagrams and layouts for the printed circuit board (PCB). The LC tank was set as 18 μH and 33 pF in this case. Bare PCB was manufactured by OSH Park (USA). All components were purchased from Digikey Electronics (USA). A detailed bill of materials was listed in tables S2 and S3. Package size for all capacitors and resistors is 0402, and 0603 for indicator light-emitting diodes. Bootloader (Adafruit Feather nRF52840 Express) was uploaded to the Bluetooth low-energy (BLE) system-on-chip by serial wire debug protocol via five reserved pads on board. Solder paste (SMDLTLFP10T5, Chip Quik) was used to attach the various surface-mount components to the PCB, facilitated by a heat gun (Int 866, Aoyue) and hot plate (MHP30, Miniware). The configuration enables wireless data transmission to any portable device using BLE protocols. The actual board size is 25 mm by 25 mm for shear pressure sensing circuit (fig. S16) and 40 mm by 40 mm for EL multiplexing circuit (fig. S17). A container was designed using commercial 3D CAD software (Autodesk Fusion 360 Version 2.0) and was printed using a stereolithography 3D printer (Form 3, Formlabs), and a textile belt (Xpand) was used to attach the container to the residual limb of a participant with lower limb amputation. The electronics and wiring are positioned outside the socket, thereby preserving the normal wearing experience and eliminating the need for socket modifications.

### Washability test

More than 30 cycles of laundry were carried out using a standard household washing machine (Whirlpool, USA). The embroidered sensors were put into a water-permeable protective sack. Each laundry cycle was conducted using 10 ml of commercial liquid detergent (Arm & Hammer, USA) at regular mode with a duration of ∼30 min, which included spinning, rinsing, and spin-drying. After each washing process, the sensors were dried overnight at room temperature.

### Human participant test

The human participants provided informed consent to participate in this study. The equipment for gait analysis was a reflective optical-marker motion capture system (OptiTrack, OR, USA) and Kistler force plates (Kistler Group, Switzerland). The research protocol was approved by the Notre Dame Institutional Review Board, protocol ID: 24-01-8293.
