# Supplementary material for: Embroidered textile sensors for real-time multiaxial force mapping in prosthetics
Source: Sci Adv. 2026 Aug 14;12(33):eaec9270. doi: 10.1126/sciadv.aec9270 (PMC13475602; doi:10.1126/sciadv.aec9270)
Supplement: Supplementary file 1 [file sciadv.aec9270_sm.pdf]

Science Advances
AAAS

Supplementary Materials for

Embroidered textile sensors for real-time multiaxial force mapping in prosthetics

Tianhao Yu *et al.*

Corresponding author: Edgar Bolívar-Nieto, ebolivar@nd.edu; Chi Hwan Lee, lee2270@purdue.edu

Sci. Adv. **12**, eaec9270 (2026)
DOI: 10.1126/sciadv.aec9270

The PDF file includes:

Figs. S1 to S17
Tables S1 to S3
Legends for movies S1 to S5

Other Supplementary Material for this manuscript includes the following:

Movies S1 to S5

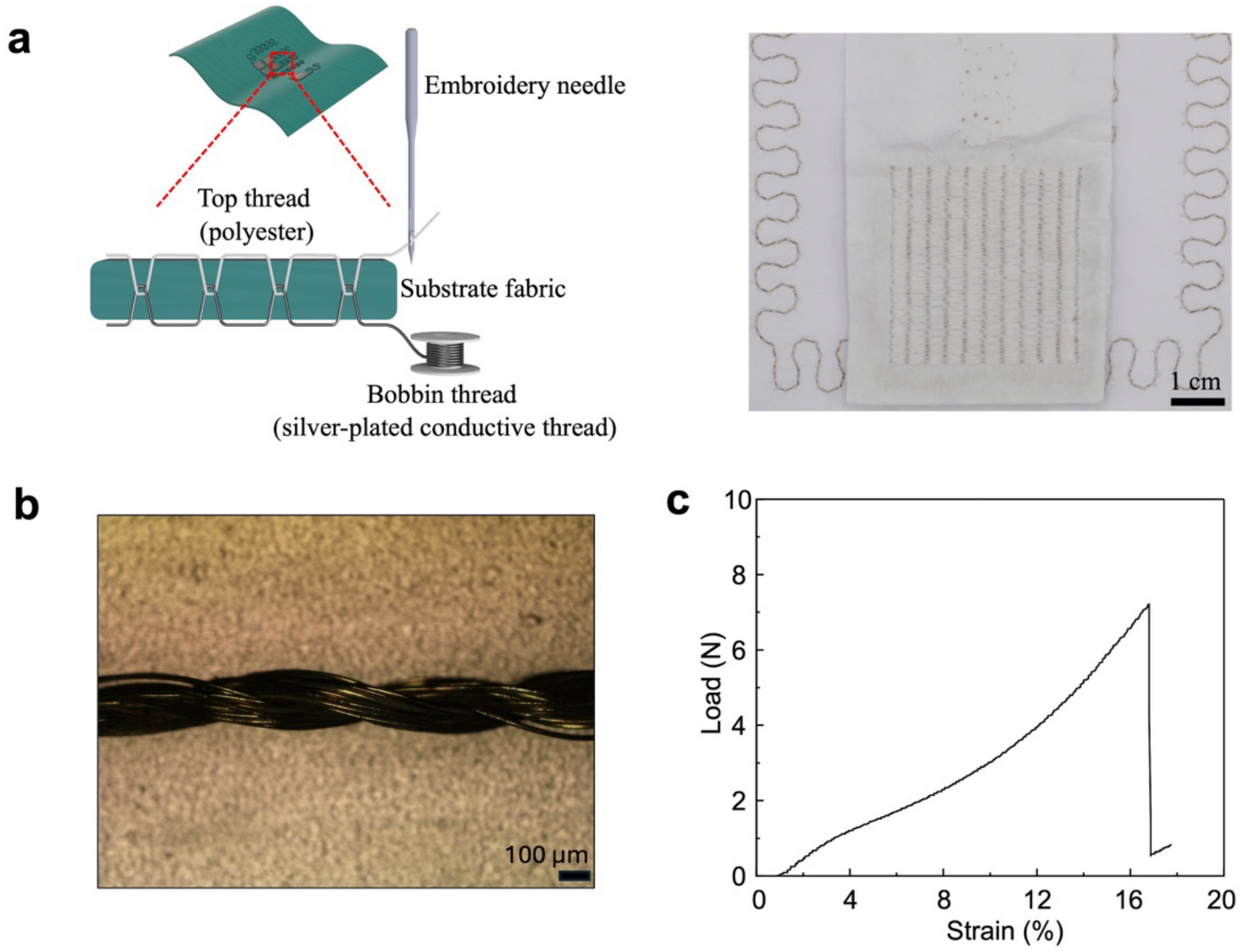

Fig. S1. (a) Schematic of the embroidery process, showing top polyester thread, silver-plated conductive bobbin thread, and substrate fabric (left) and an assembled sensor (right). **(b)** Optical picture of the conductive thread. **(c)** Strain-load curve of the conductive thread.

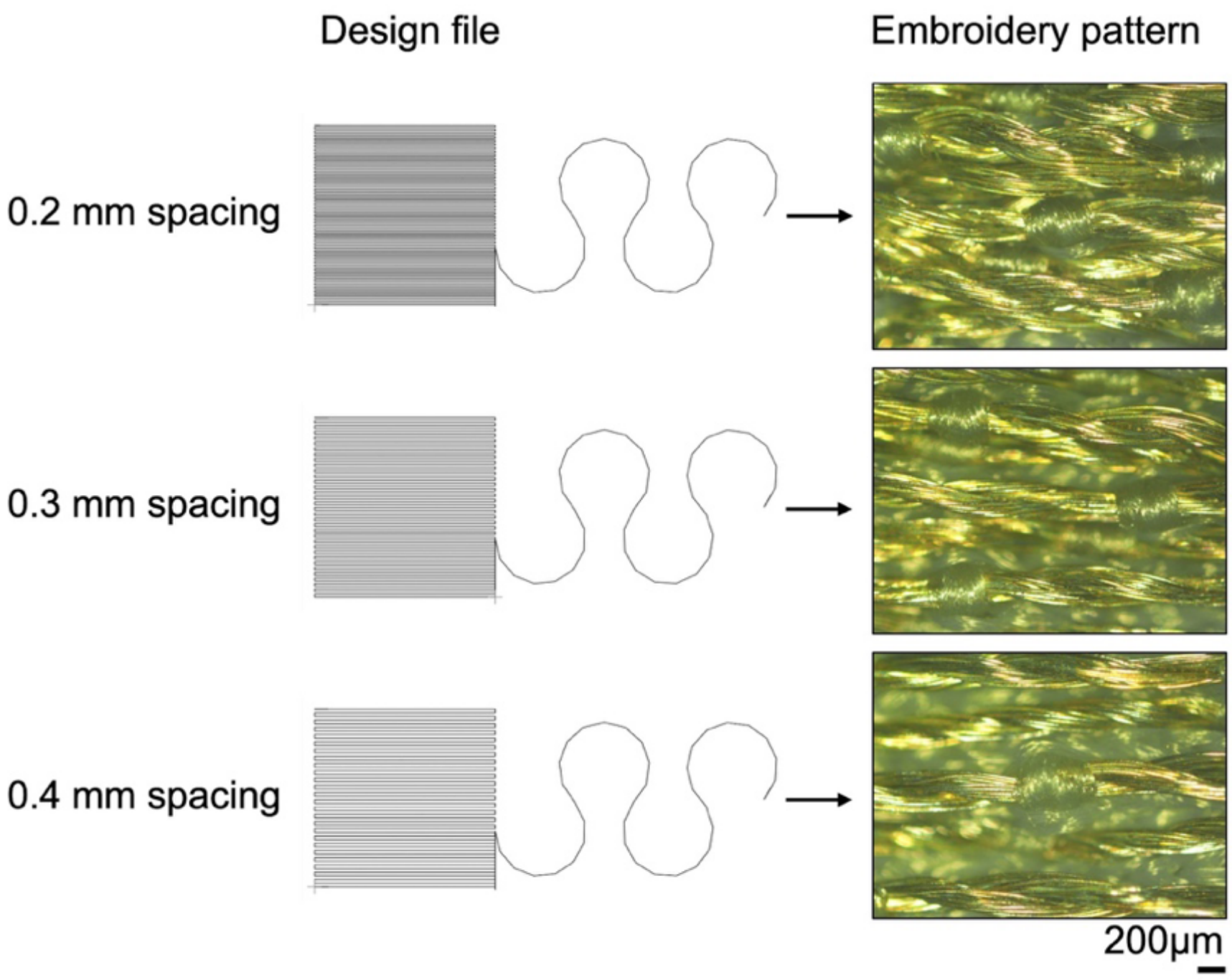

Fig. S2. Different spacing distance between embroidered lines.

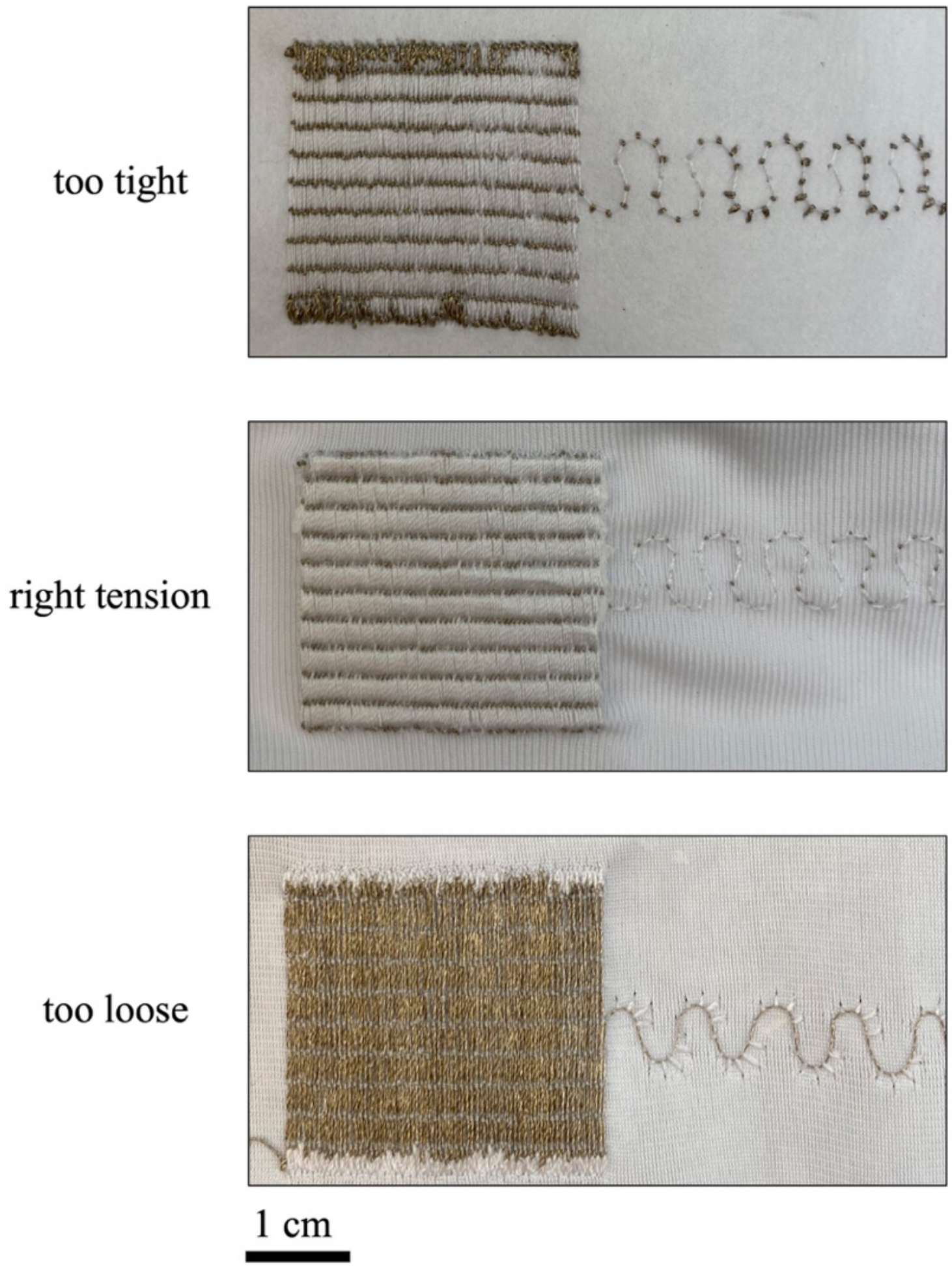

Fig. S3. Different embroidery tension conditions.

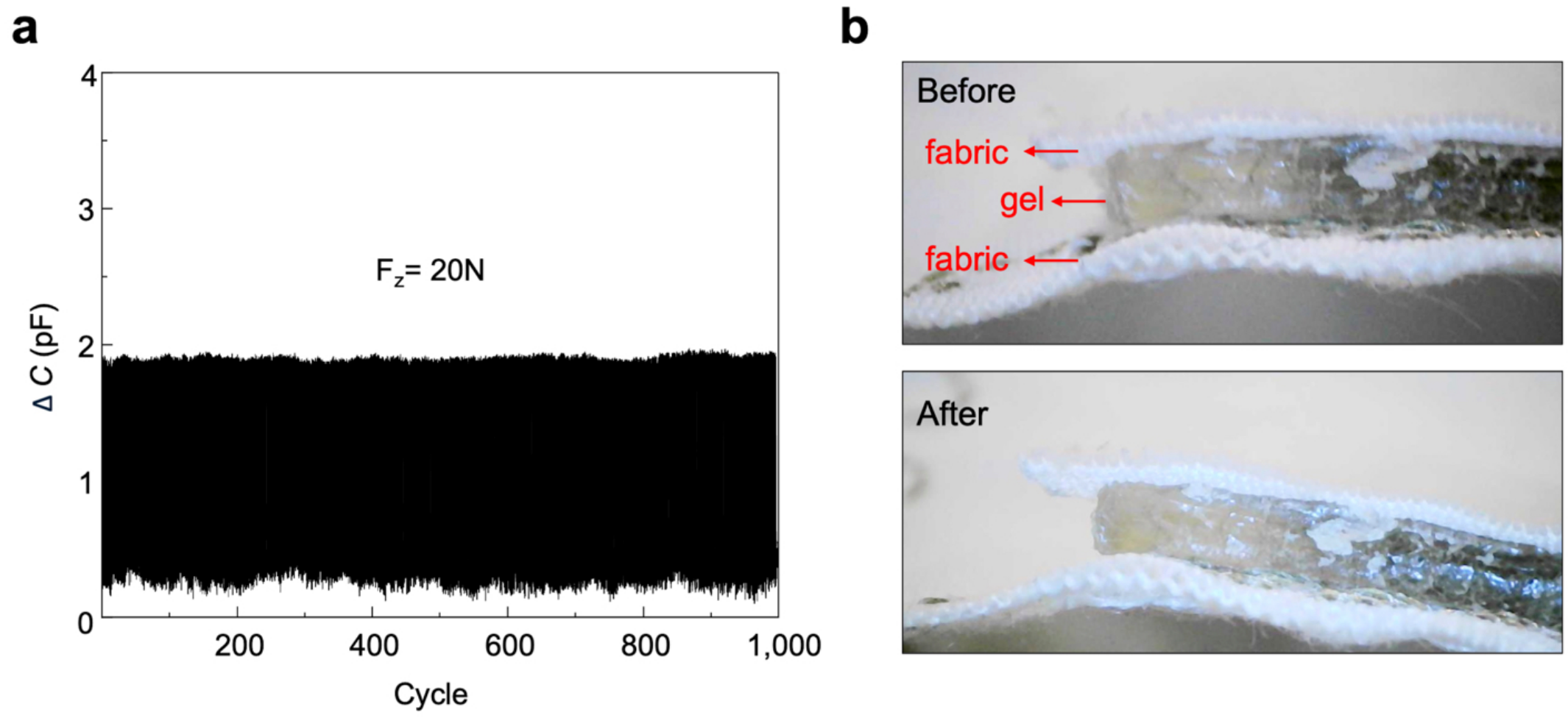

Fig. S4. (a) Cyclic shear test. **(b)** Cross-sectional photographs before and after the test.

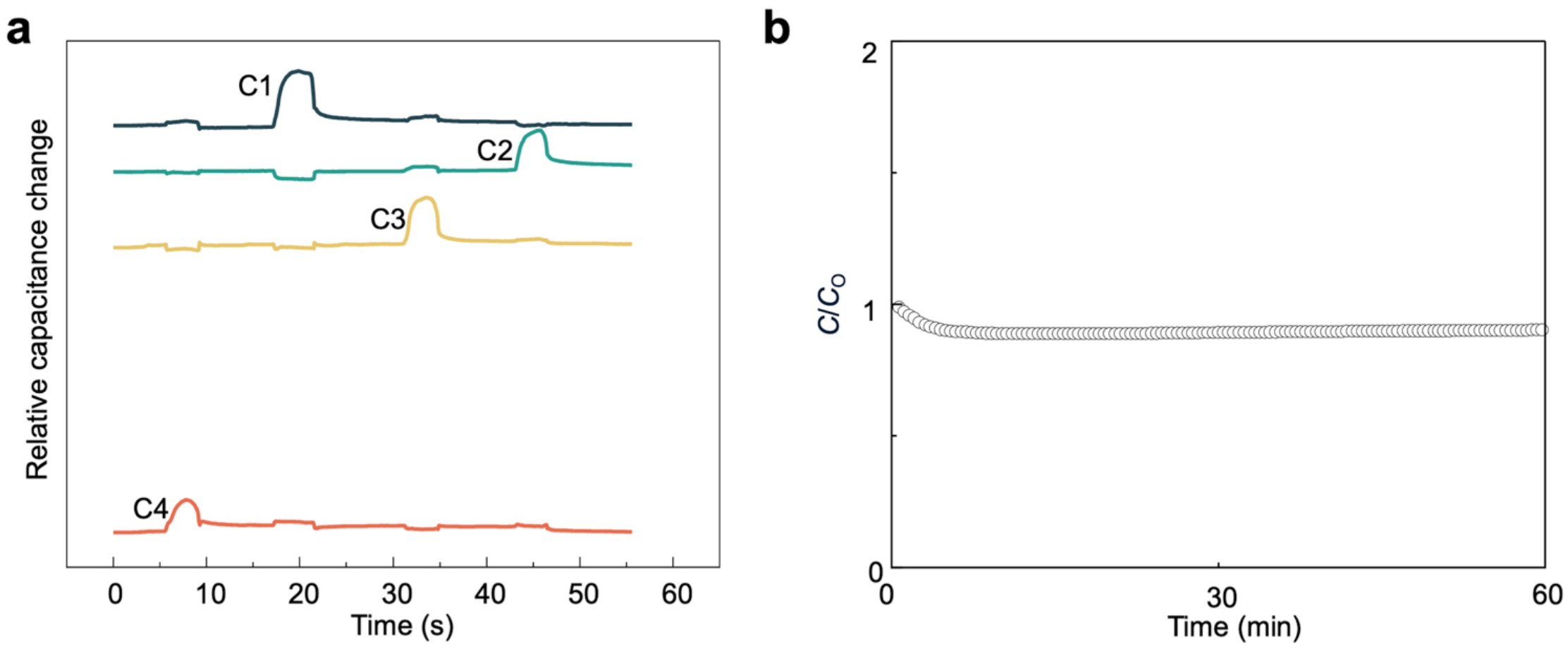

Fig. S5. (a) Cross-talk characterization. **(b)** Long-term drift test.

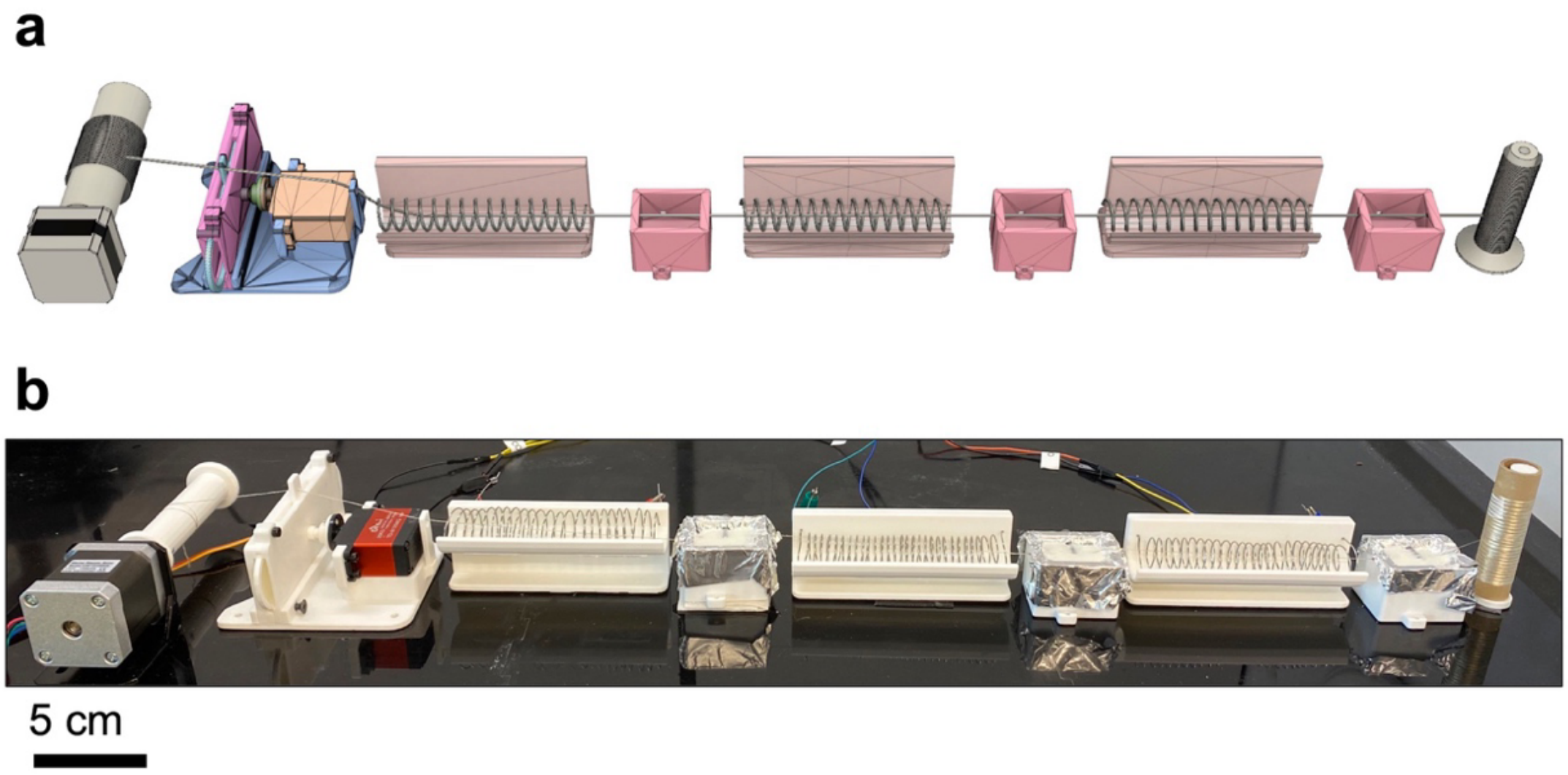

Fig. S6. (a) Rendering image of the coating system. **(b)** Real image of the coating system.

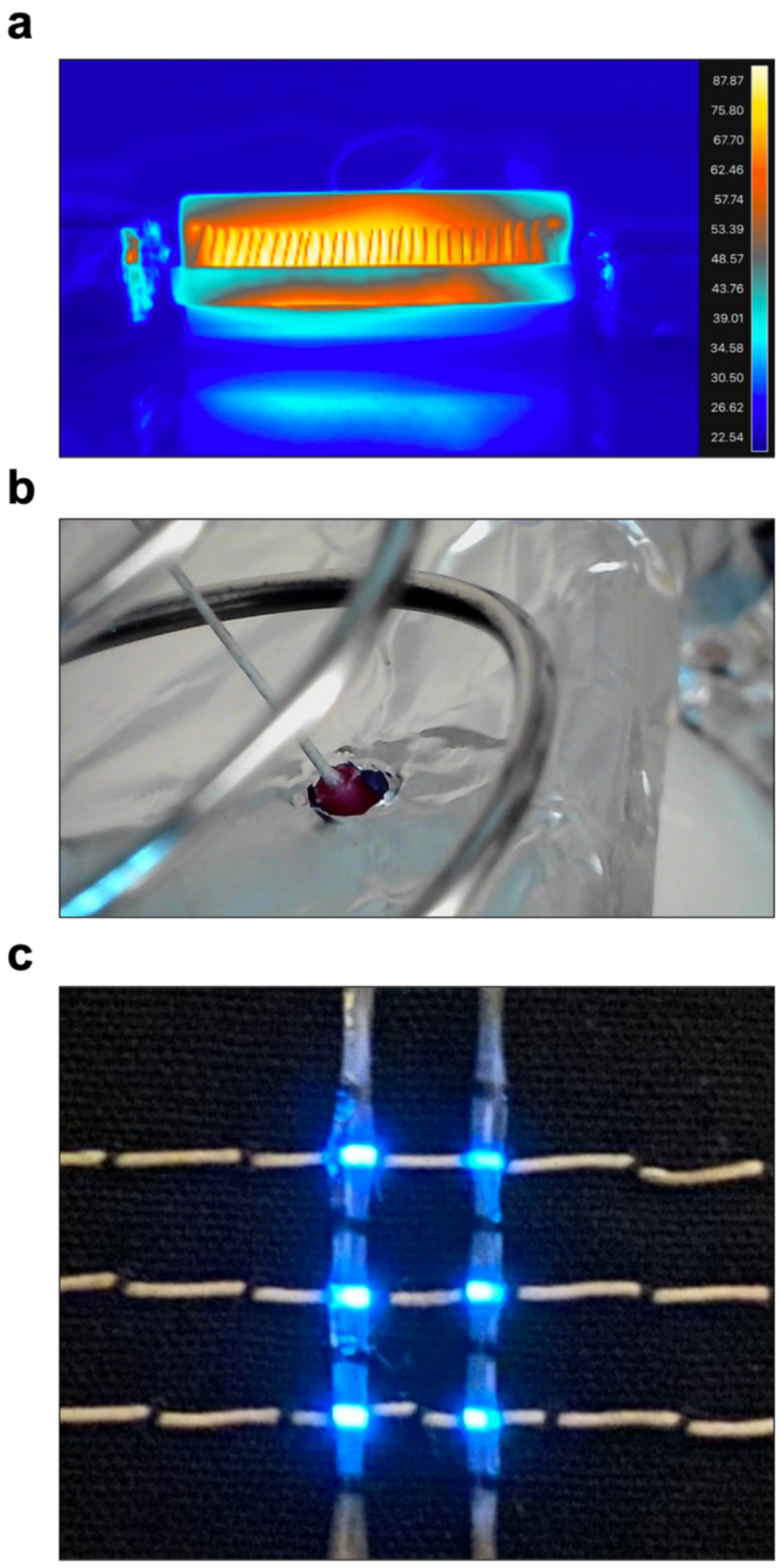

Fig. S7. (a) Thermal image of the heating coil. **(b)** Coated thread coming out from the nozzle. **(c)** Textile display encapsulated with fabric glue.

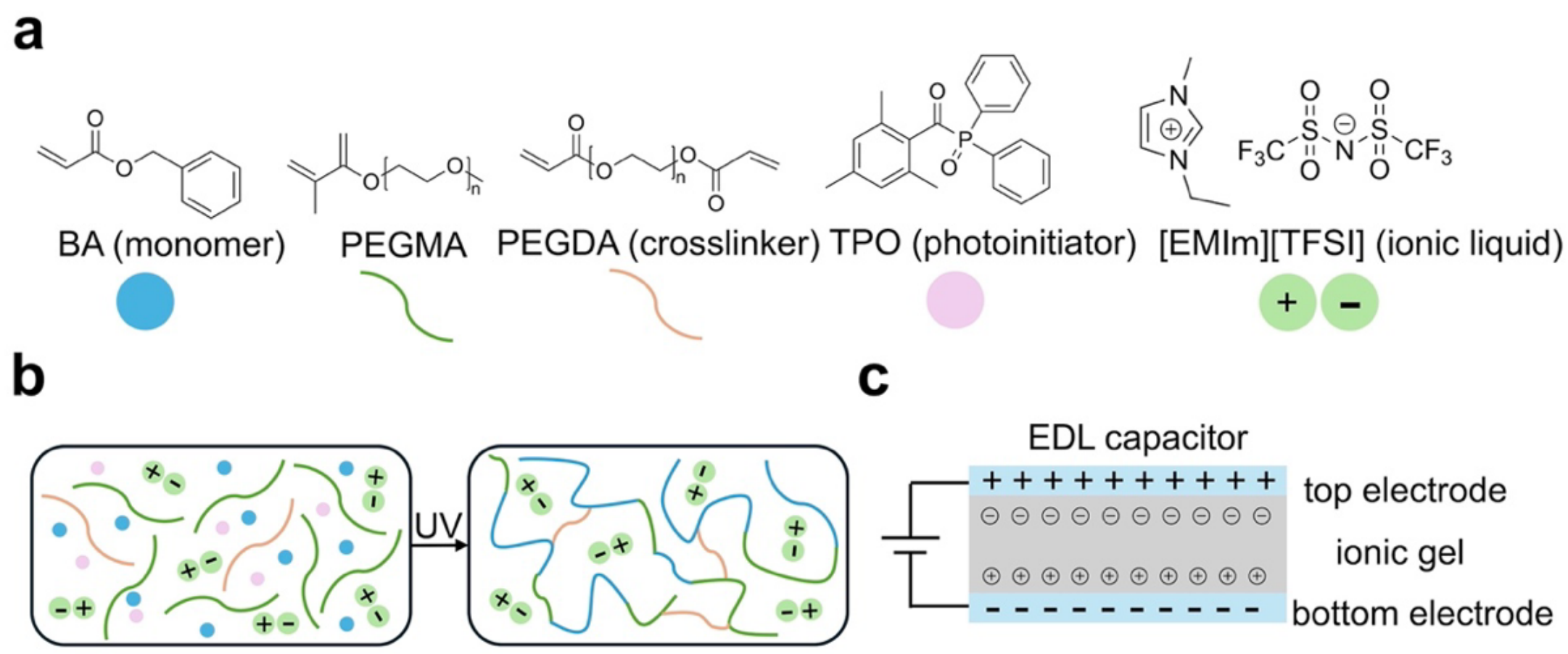

Fig. S8. (a) Detailed chemical structures of BA, PEGMA, PEGDA, TPO and [EMIm][TFSI] that are used to prepare the ionic gel precursor solution. **(b)** Illustrations of the photopolymerization process during photopolymerization. **(c)** Sensing mechanism of the electric double layer capacitor.

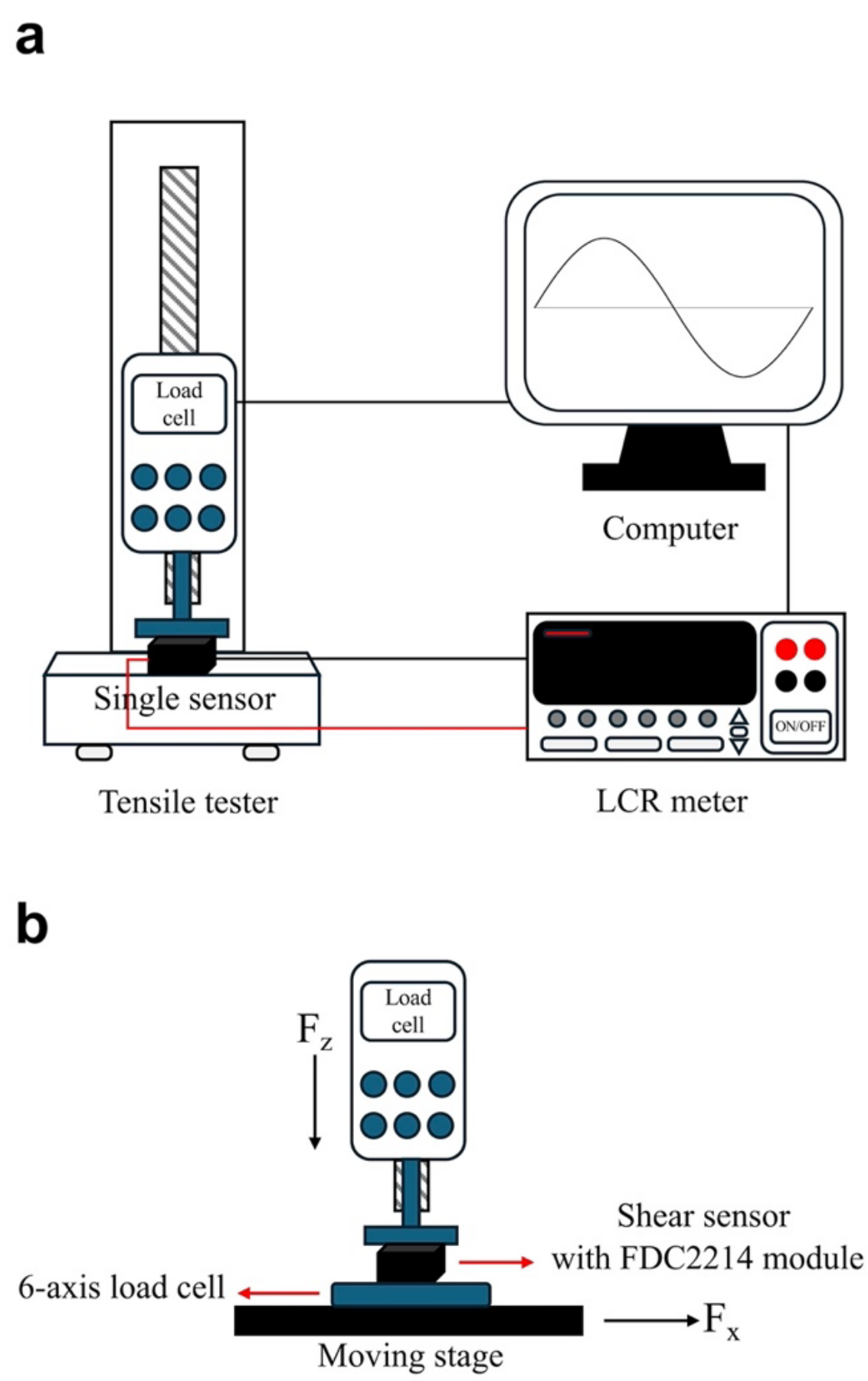

Fig. S9. Schematic of the electromechanical test setup. **(a)** Normal pressure. **(b)** Shear pressure.

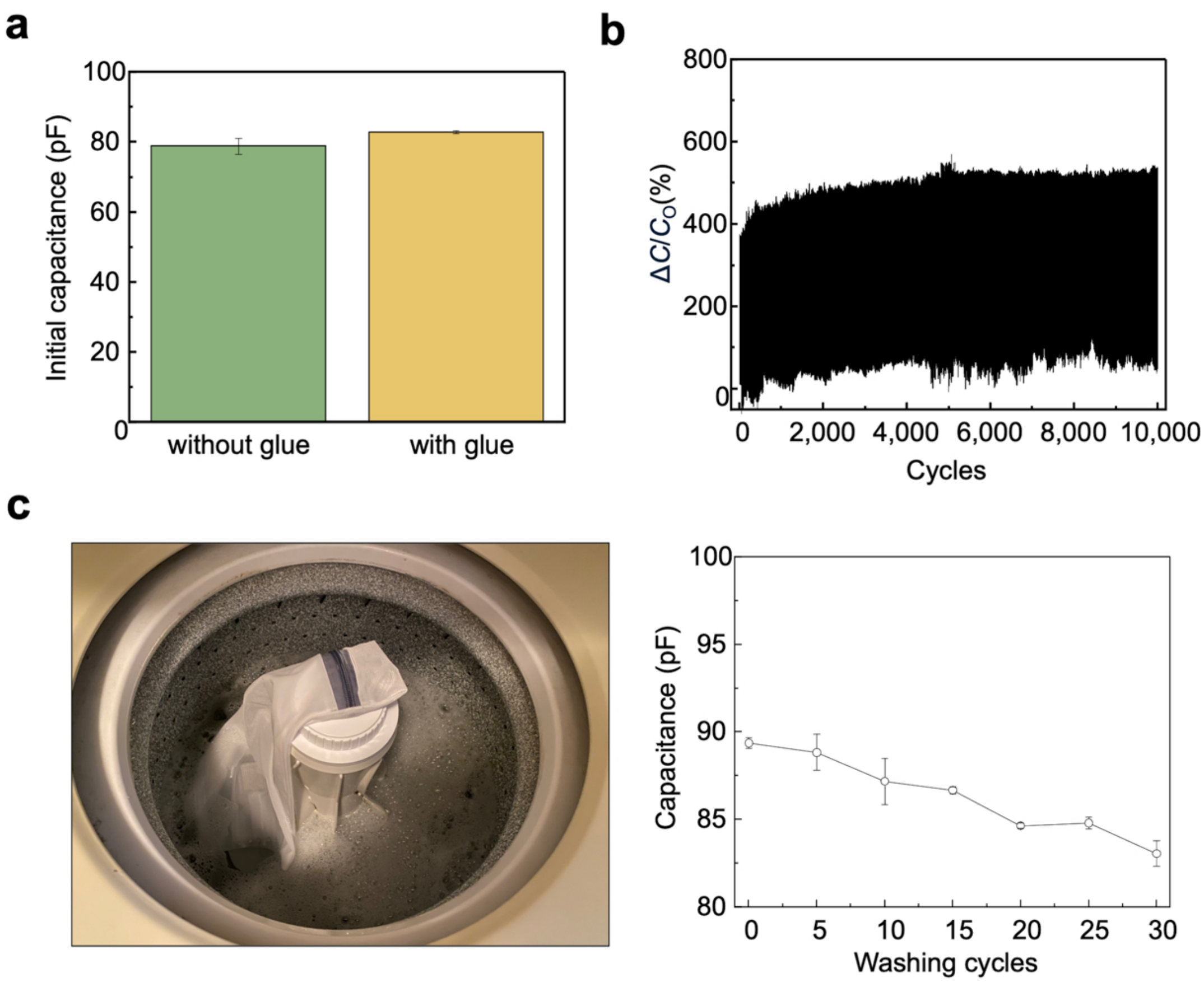

Fig. S10. (a) Initial capacitance with/without glue combing embroidered electrodes and ionic gel. **(b)** Relative capacitance change without glue through 10,000 loading/unloading cycles at a pressure of 500 kPa. c) Washability test.

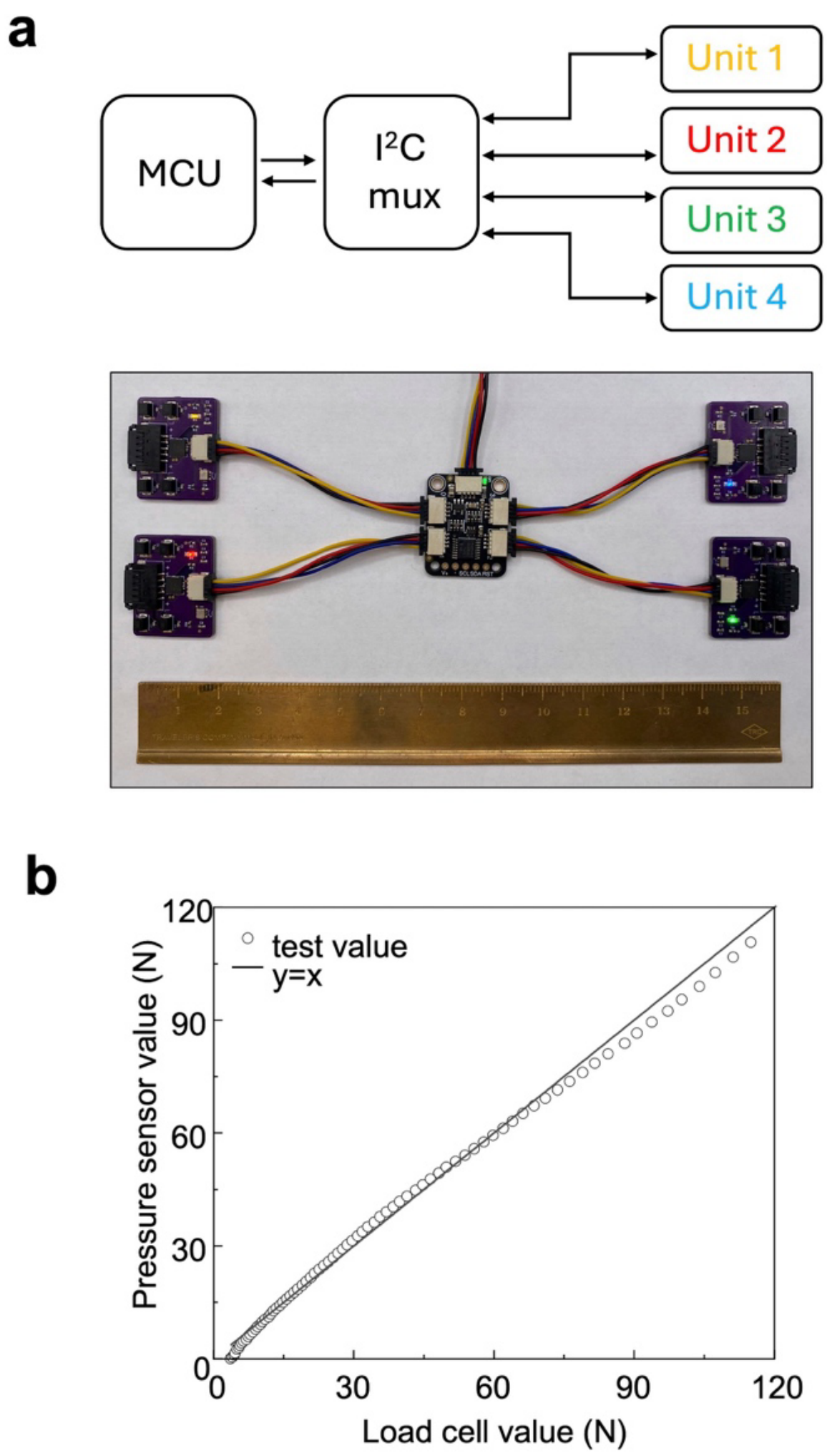

Fig. S11. **(a)** FDC2214 units connected via a I^2c mux. **(b)** Calibration result.

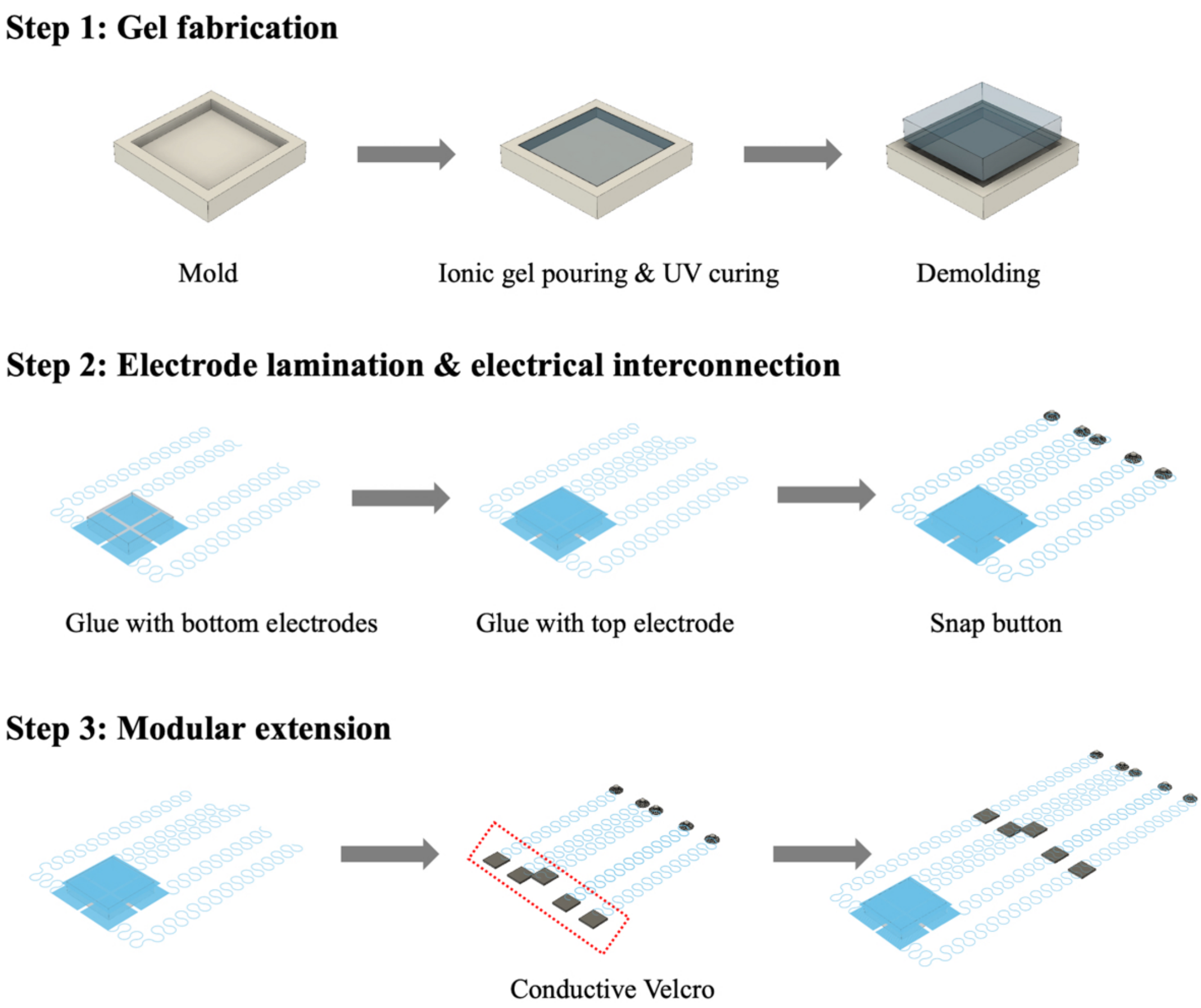

Fig. S12. Fabrication process of the multiaxial pressure sensor.

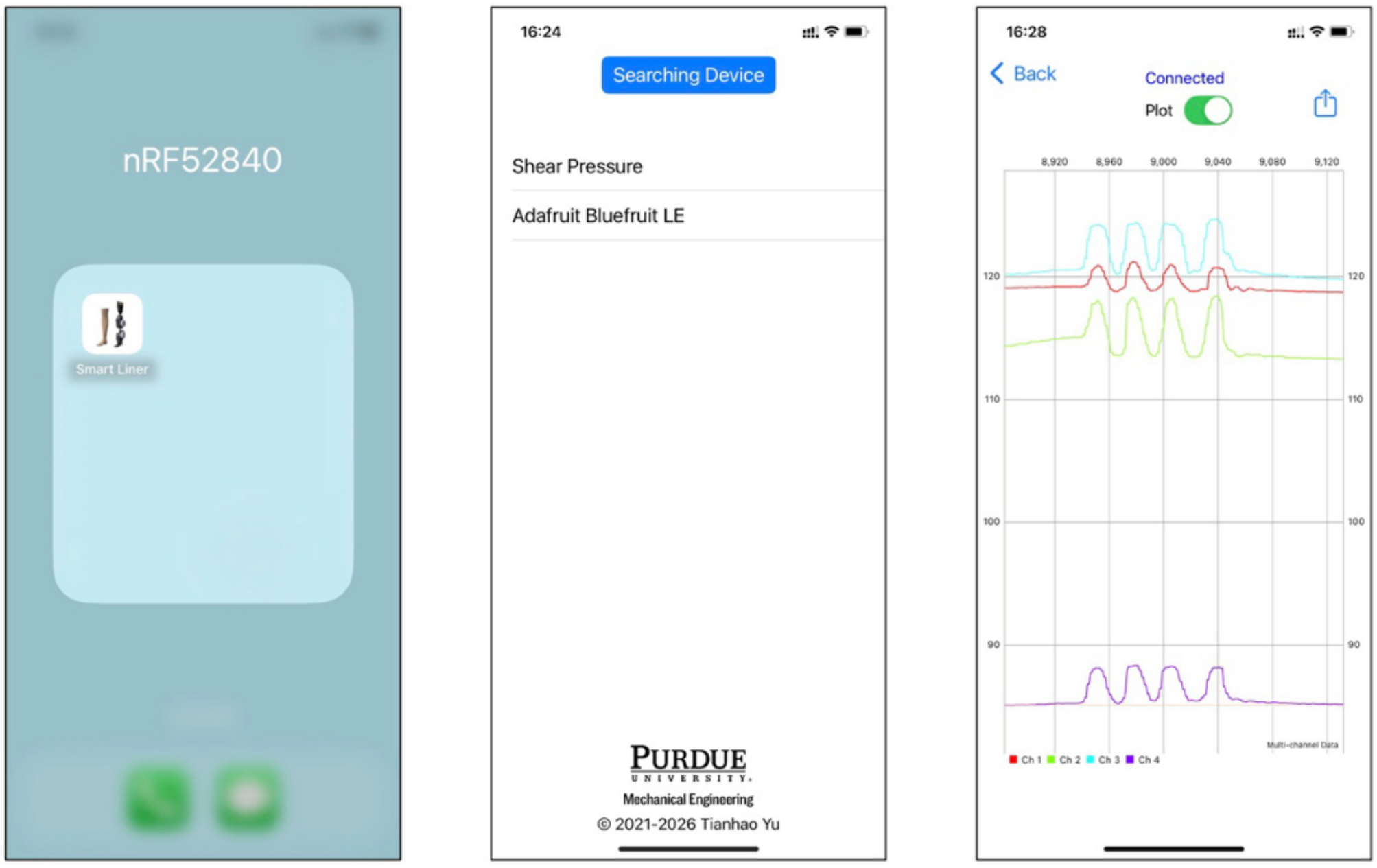

Fig. S13. Customized iOS application for the wireless DAQ device.

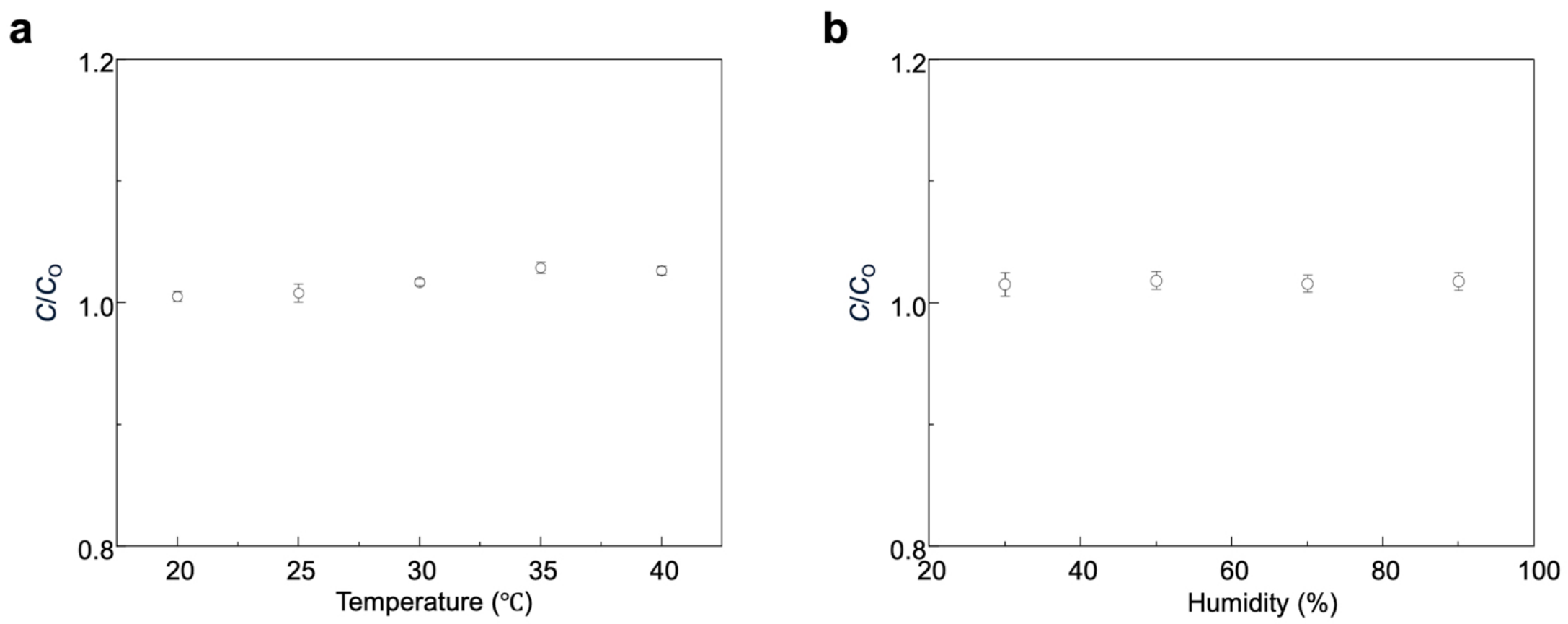

Fig. S14. (a) Normalized capacitance under different temperatures. Error bars represent standard deviation, n = 3 for each group. **(b)** Normalized capacitance under different humidities. Error bars represent standard deviation, n = 3 for each group.

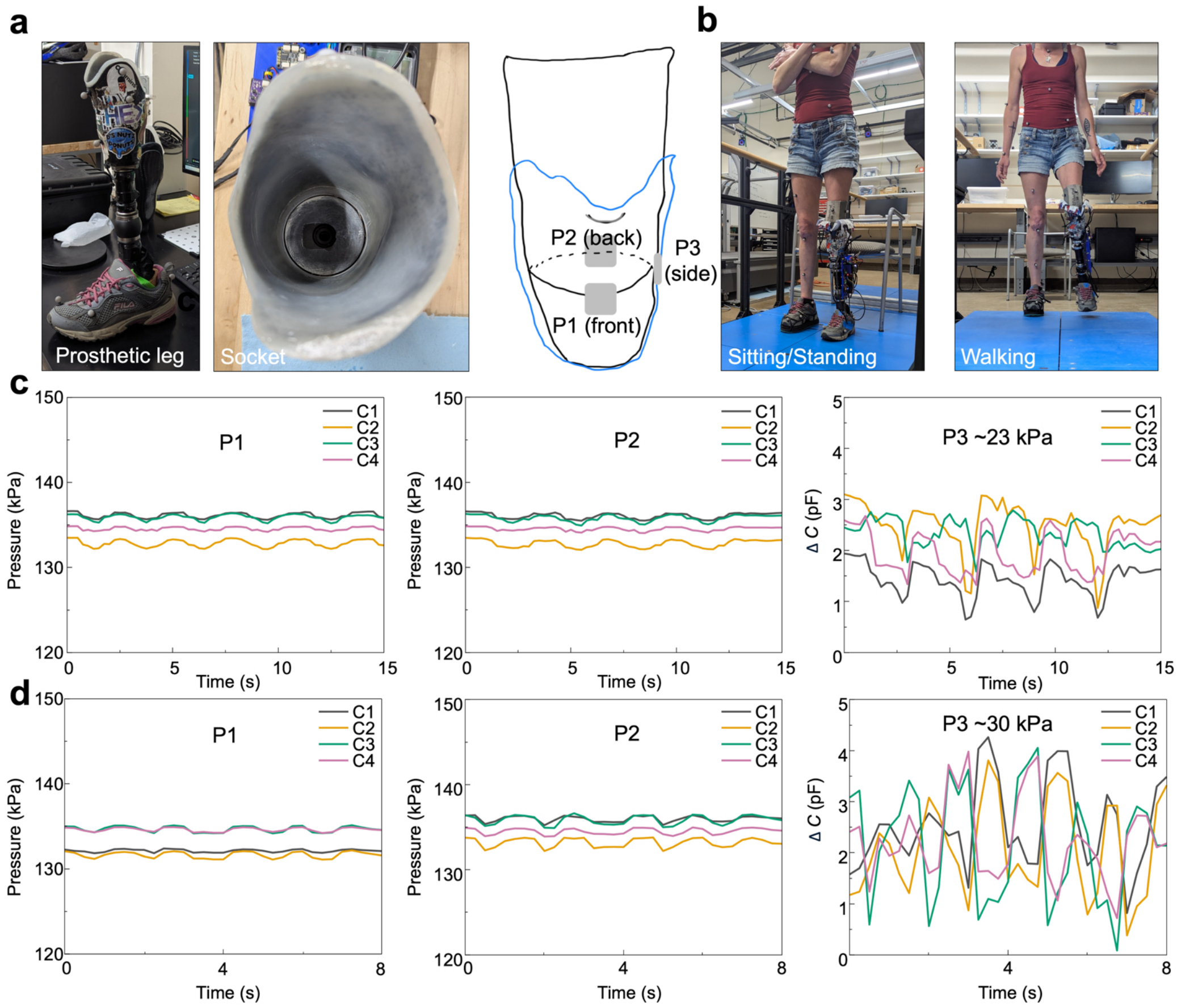

Fig. S15. In vivo evaluation of the system in a female individual with transtibial amputation. (a) Photographs of the prosthetic leg with three sensors attached inside the socket, P1, P2 and P3. (b) Test scenarios including sitting, standing, and walking. (c) Recorded normal pressures from P1, P2 and shear pressure from P3 during sit and stand. (d) Recorded normal pressures from P1, P2 and shear pressure from P3 during walking.

a

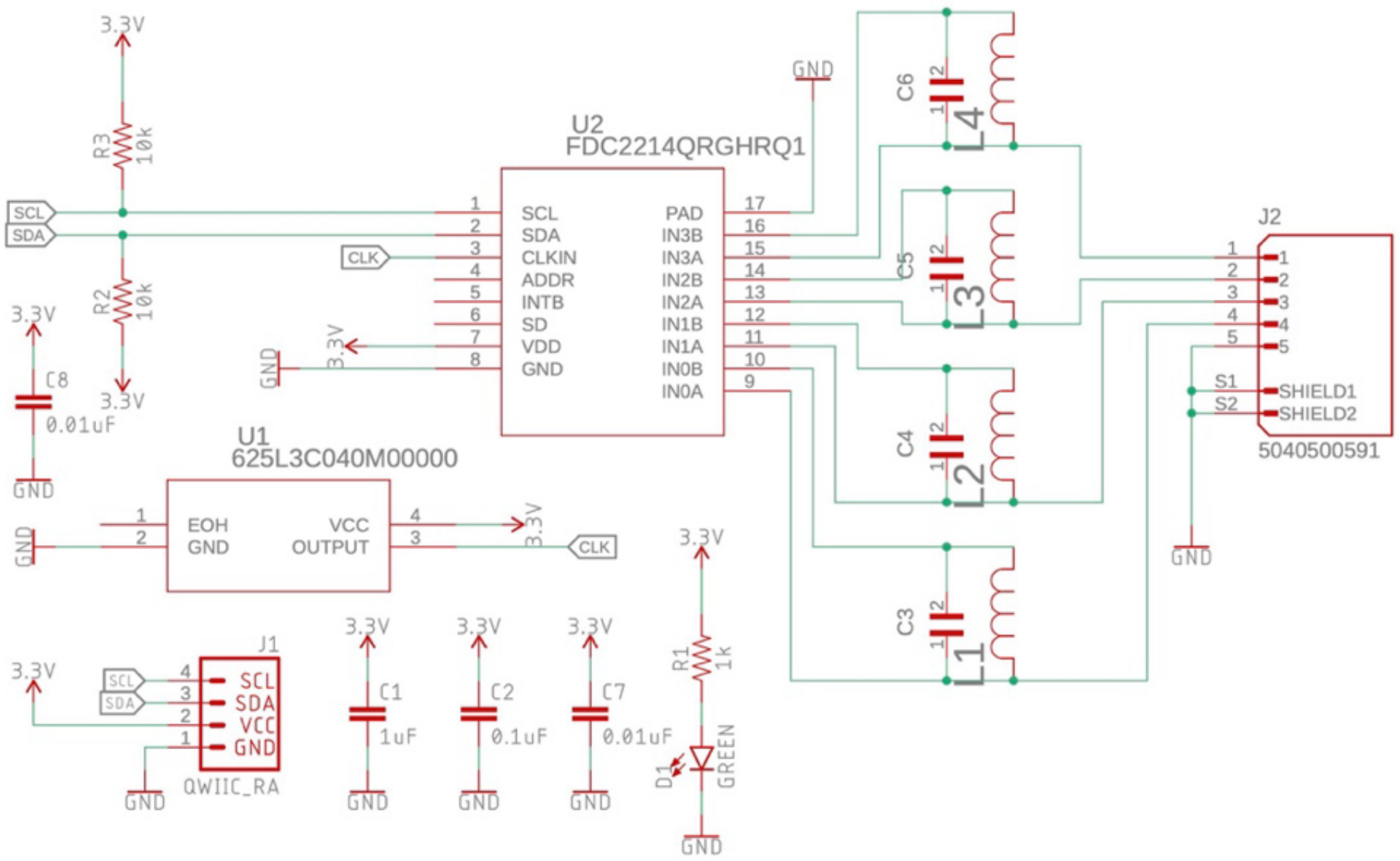

b

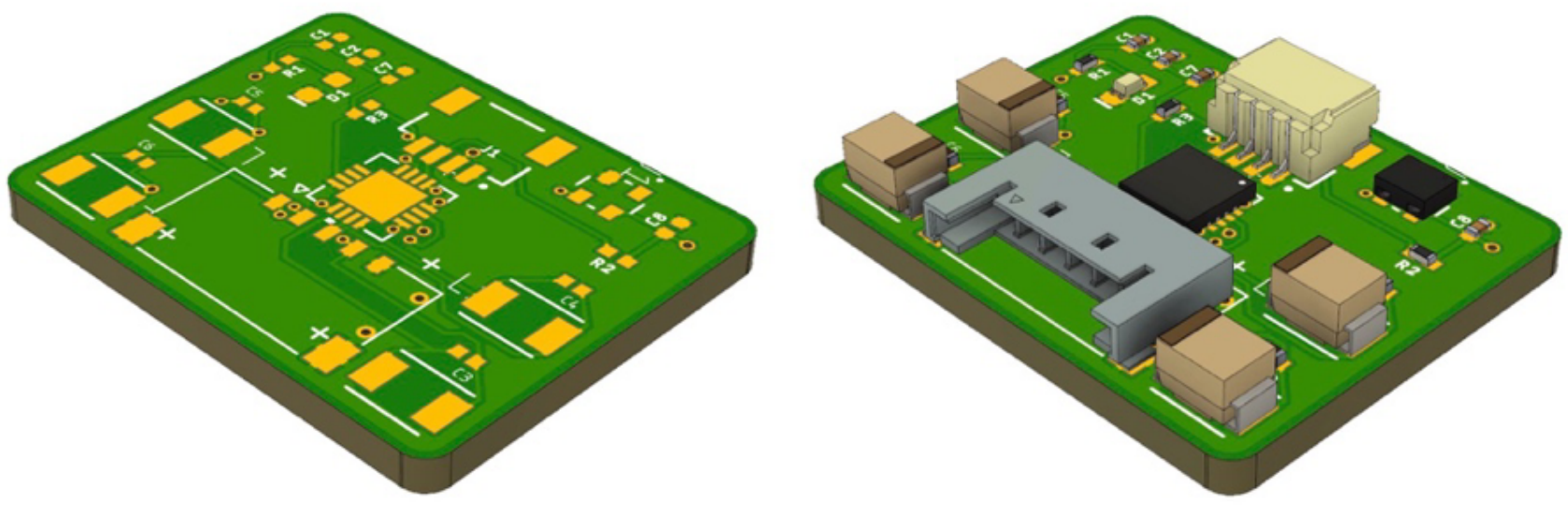

Fig. S16. (a) Schematic of the measuring system. **(b)** PCB assembling (25 mm x 20 mm).

a

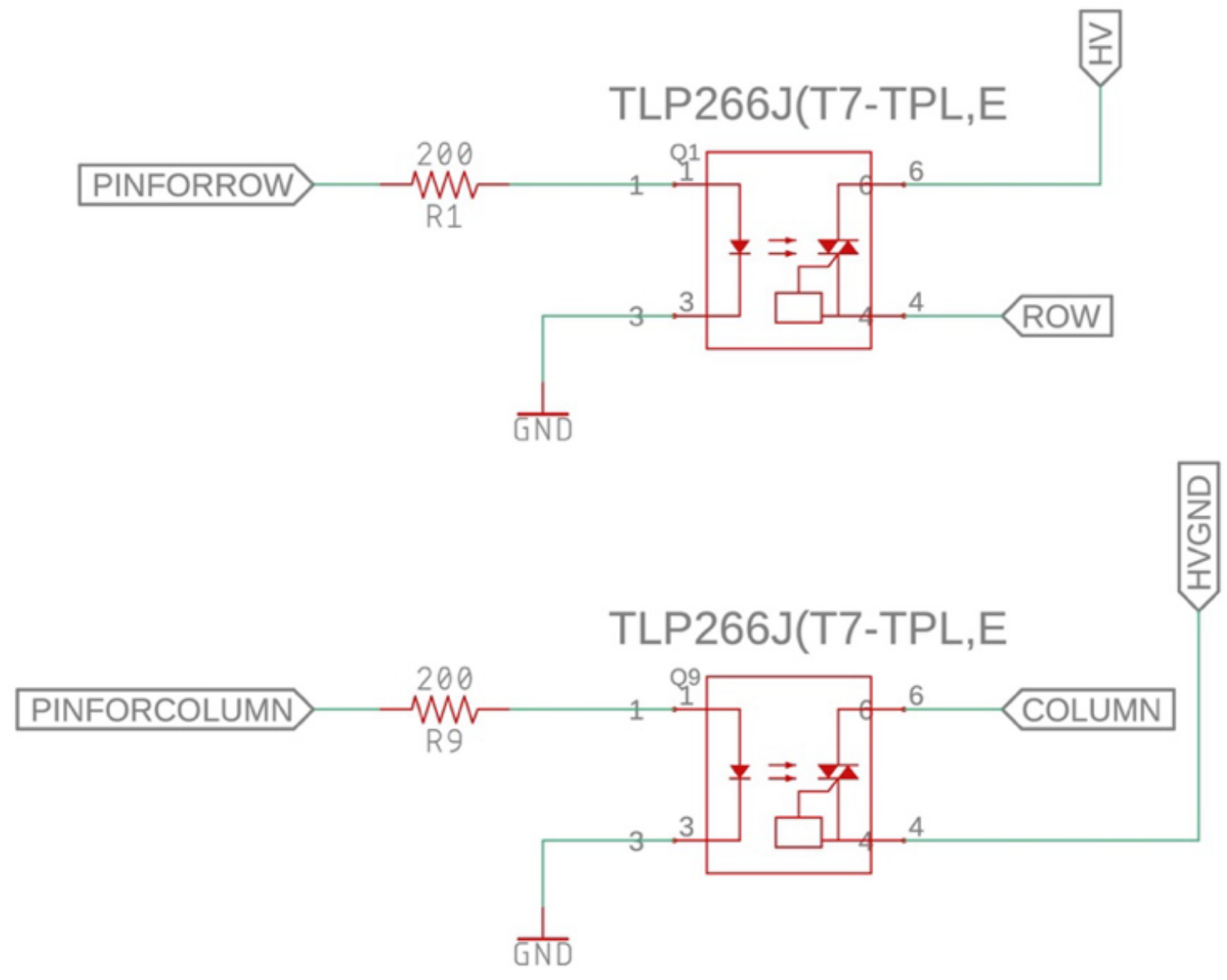

b

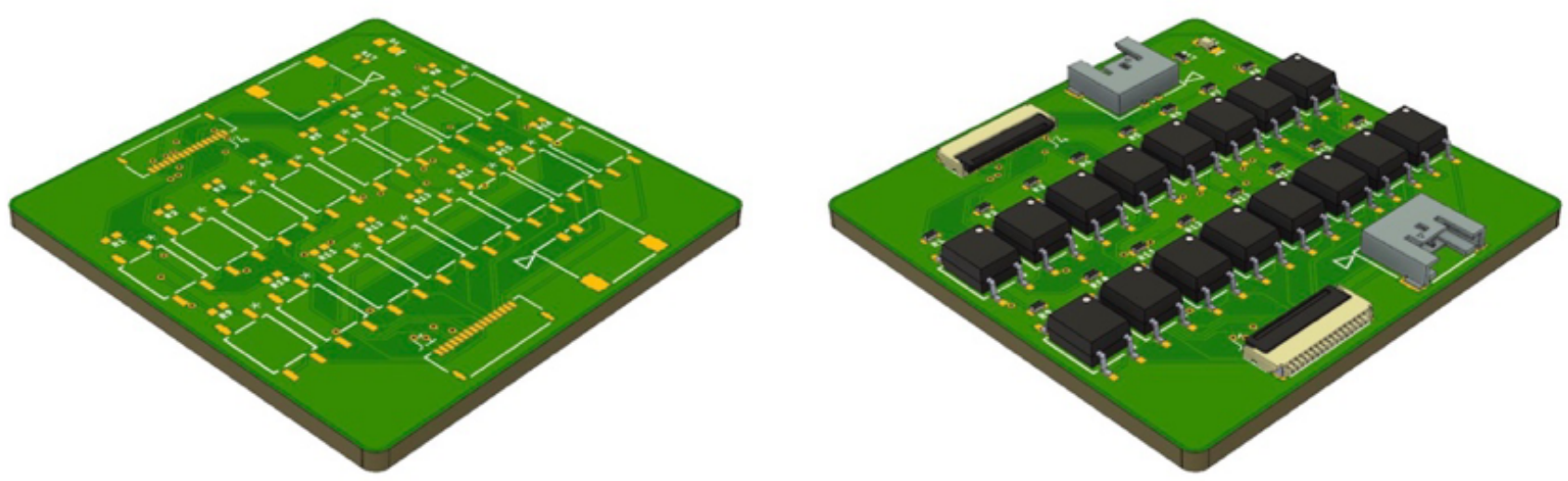

Fig. S17. (a) Schematic of the textile display multiplexing system. **(b)** PCB assembling (40 mm x 40 mm).

Year	Ref	Material	Type	Channel	Upper Detection Limit
2026	This work	Textile and ionic gel	Capacitive	Modular design, as many as possible	500 kPa
2024	23	3d-printed polyurethane	Capacitive	4	400 kPa
2024	45	Micro-camera	Light-based	6	22 N
2023	46	Copper and PDMS	Capacitive	64	300 kPa
2022	47	Liquid metal	Capacitive	2	20 N
2022	24	Porous PDMS	Capacitive	4	500 kPa
2020	48	PVDF-HFP	Resistive/capacitive	1	100 kPa
2020	49	Copper coil	Inductance	64	105N

Table S1. Comparison of multiaxial pressure sensors in literature that can sense both normal and shear forces.

Components	Manufacturer	Part number
IC capacitive sensing	Texas Instruments	FDC2214QRGHRQ1
External oscillator	CTS Components	625L3C040M00000
Inductor (18 μH)	Bourns Electronics	CMH322522-180KL
Capacitor (33 pF)	Murata Electronics	GJM1555C1H330FB01D
I^2C connector	JST	SM04B-SRSS-TB
Four channels connector	Molex	5040500591

Table S2. Bill of materials (shear pressure sensing circuit).

Components	Manufacturer	Part number
Solid state relay	Toshiba	TLP266J(T7-TPL,E
Current-limiting resistor	Panasonic	0.1% 1/16W 0402
Connector1	Molex	5034801600
Connector2	Molex	5040500291

Table S3. Bill of materials (EL multiplexing circuit).

Movie S1. Fabrication process of the electrodes.

Movie S2. Ionic gel deformation upon normal and shear pressure.

Movie S3. Automatic fabrication process of the EL thread.

Movie S4. Textile display sweeping.

Movie S5. Dynamic gait analysis in an individual with transtibial amputation.
